# Loss of E-cadherin provides tolerance to centrosome amplification in epithelial cancer cells

**DOI:** 10.1083/jcb.201704102

**Published:** 2018-01-02

**Authors:** Alexander D. Rhys, Pedro Monteiro, Christopher Smith, Malti Vaghela, Teresa Arnandis, Takuya Kato, Birgit Leitinger, Erik Sahai, Andrew McAinsh, Guillaume Charras, Susana A. Godinho

**Affiliations:** 1 Barts Cancer Institute–CRUK Centre, Queen Mary University of London, John Vane Science Centre, London, England, UK; 2 Centre for Mechanochemical Cell Biology, Division of Biomedical Science, Warwick Medical School, University of Warwick, Coventry, England, UK; 3 London Centre for Nanotechnology, University College London, London, England, UK; 4 Tumour Cell Biology Laboratory, Francis Crick Institute, London, England, UK; 5 Molecular Medicine Section, National Heart and Lung Institute, Imperial College London, London, England, UK

## Abstract

Centrosome amplification is a common feature of human tumors. To survive, cancer cells cluster extra centrosomes during mitosis, avoiding the detrimental effects of multipolar divisions. However, it is unclear whether clustering requires adaptation or is inherent to all cells. Here, we show that cells have varied abilities to cluster extra centrosomes. Epithelial cells are innately inefficient at clustering even in the presence of HSET/KIFC1, which is essential but not sufficient to promote clustering. The presence of E-cadherin decreases cortical contractility during mitosis through a signaling cascade leading to multipolar divisions, and its knockout promotes clustering and survival of cells with multiple centrosomes. Cortical contractility restricts centrosome movement at a minimal distance required for HSET/KIFC1 to exert its function, highlighting a biphasic model for centrosome clustering. In breast cancer cell lines, increased levels of centrosome amplification are accompanied by efficient clustering and loss of E-cadherin, indicating that this is an important adaptation mechanism to centrosome amplification in cancer.

## Abstract (precis)

Centrosome clustering is essential for the survival of cells containing supernumerary centrosomes. Rhys et al. show that centrosome clustering is a two-step mechanism in which increased cortical contractility, driven by loss of E-cadherin, restricts centrosome movement, facilitating HSET-mediated clustering.

## Introduction

The presence of supernumerary centrosomes is a hallmark of human tumors (Zyss and Gergely, 2009; Chan, 2011). Recent work has shown that these abnormalities can accelerate and promote tumorigenesis in vivo, induce aneuploidy, and promote cell invasion (Ganem et al., 2009; Silkworth et al., 2009; Godinho et al., 2014; Coelho et al., 2015; Serçin et al., 2016; Levine et al., 2017). However, the presence of extra centrosomes presents a burden for cells, as they need to overcome the detrimental effects of multipolar divisions to avoid death (Kwon et al., 2008; Ganem et al., 2009). To date, centrosome clustering, defined as the close association of extra centrosomes during mitosis allowing the formation of a pseudo-bipolar spindle, is the best-characterized mechanism of coping with extra centrosomes (Brinkley, 2001; Marthiens et al., 2012; Godinho and Pellman, 2014). Most cancer cell lines with high levels of centrosome amplification (as defined by >30% of cells containing extra centrosomes) are highly proficient at clustering extra centrosomes (Ring et al., 1982; Quintyne et al., 2005; Kwon et al., 2008; Ganem et al., 2009). Previous work described factors important for centrosome clustering, including proteins involved in the spindle assembly checkpoint and microtubule motors associated with the mitotic spindle, such as HSET/KIFC1 (Quintyne et al., 2005; Basto et al., 2008; Kwon et al., 2008; Leber et al., 2010). In addition, cortical actin was shown to play a key role in this process by providing spatial cues that guide centrosomes via astral microtubules, a process that seems to depend on the unconventional myosin Myo10 and actomyosin contractility (Kwon et al., 2008, 2015). Still, the prevalence and efficiency of each of the clustering mechanisms in transformed and nontransformed cells remains unknown.

During interphase, cortical contractility is regulated by E-cadherin at the adherens junctions (AJs), the major sites of cell–cell adhesion in epithelial cells (Takeichi, 2014). The presence of E-cadherin and the establishment of AJs are essential for the generation of cortical tension important for tissue homeostasis (Priya and Yap, 2015). However, it has also been reported that the presence of E-cadherin at the AJs triggers a signaling cascade leading to a local decrease in cortical contractility via down-regulation of the small GTPase RhoA activity (Hidalgo-Carcedo et al., 2011; Hendrick et al., 2016). Depletion of the GTPase-activating proteins (GAPs) p190RhoGAP and DLC3, which negatively regulate RhoA (Jaffe and Hall, 2005), led to increased contractility and AJ destabilization (Hidalgo-Carcedo et al., 2011; Hendrick et al., 2016). The discoidin domain receptor 1 (DDR1), which localizes to the AJs in an E-cadherin–dependent manner, was shown to recruit p190RhoGAP to inhibit contractility at the sites of cell–cell adhesion (Hidalgo-Carcedo et al., 2011). Depletion of DDR1 leads to a RhoA-ROCK–dependent increase of actomyosin contractility at the sites of cell–cell adhesion, resulting in loss of cell–cell cohesion and defective collective cell migration (Hidalgo-Carcedo et al., 2011).

Here we revealed that centrosome clustering efficiency depends on the cell type. We found that epithelial cells have low clustering efficiency and do not tolerate extra centrosomes. Loss of E-cadherin or DDR1 is sufficient to promote centrosome clustering through increased cortical contractility. Centrosome tracking during mitosis showed that cortical contractility restricts centrosome movement at a distance required to enable HSET-mediated centrosome clustering. Thus, we propose a two-step model for centrosome clustering in which the close proximity of centrosomes caused by actomyosin contractility precedes HSET-mediated centrosome clustering. Consequently, the loss of E-cadherin restores viability of epithelial cells containing extra centrosomes, and this loss is observed in breast cancer cell lines with higher levels of centrosome amplification. We propose that E-cadherin loss is important for the proliferation and survival of cancer cells with extra centrosomes.

## Results

### Nontransformed cells cluster supernumerary centrosomes with varied efficiency

**Figure 1.**
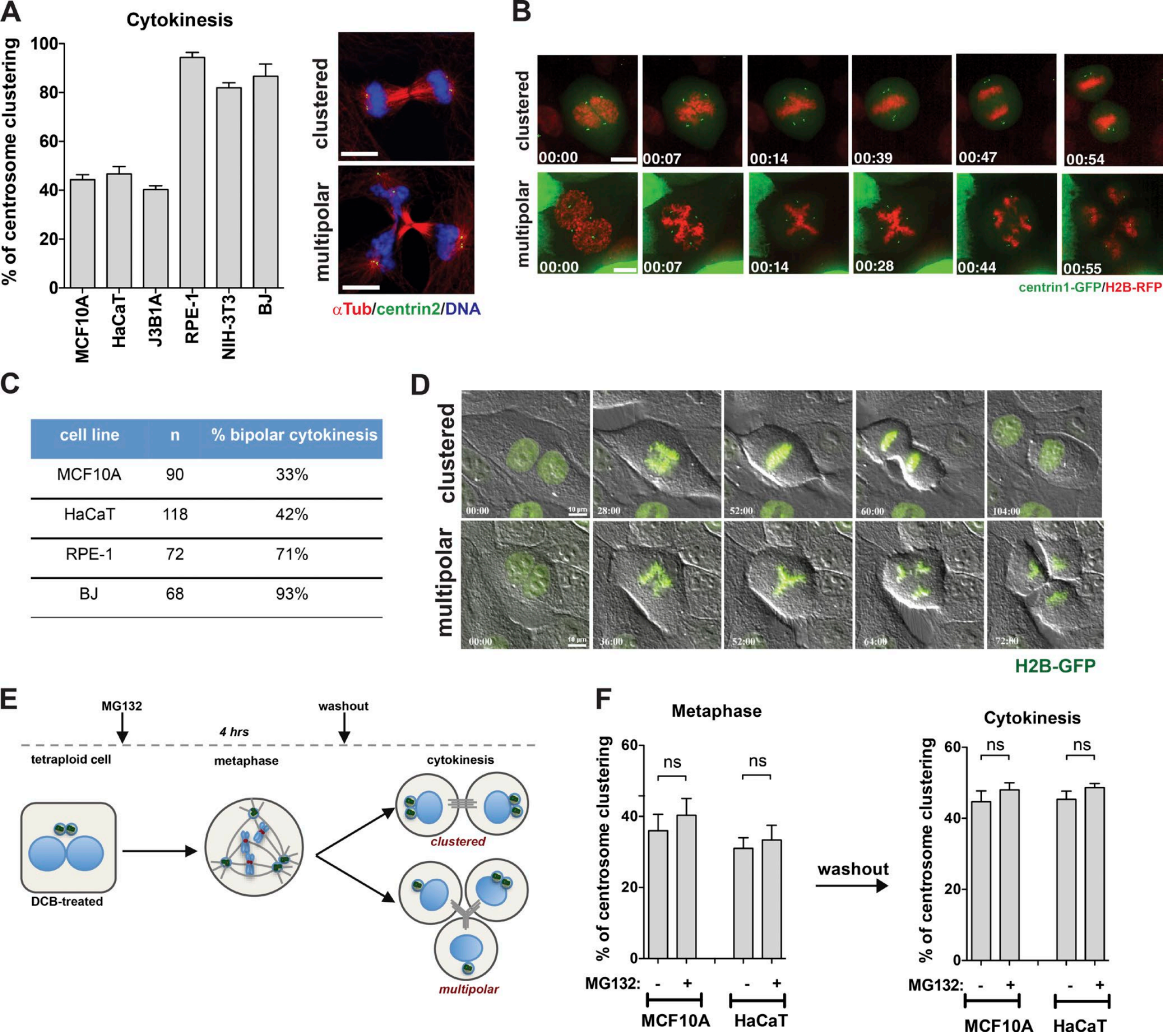
**Nontransformed cells exhibit varied ability to cluster supernumerary centrosomes.** (A, left) Quantification of centrosome clustering in tetraploid cells at cytokinesis (*n* = 150). (Right) Images depicting examples of cells in cytokinesis with extra centrosomes: bipolar clustered and multipolar. Cells were stained for microtubules (α-Tub, red), centrioles (centrin2, green), and DNA (blue). (B) Images from videos of tetraploid MCF10A cells expressing centrin1-GFP and H2B-RFP. Time scale: hours:minutes. (C) Quantification of cells undergoing bipolar divisions by live-cell imaging. (D) Images from videos of tetraploid MCF10A cells expressing H2B-GFP. (E) Schematic representation of the proteasome inhibitor (MG132) treatment and washout. (F) Quantification of centrosome clustering in cells treated with 10 µM MG132 (4 h) at metaphase (*n* = 300) and cytokinesis (*n* = 150). For all graphics, error bars represent mean ± SD from three independent experiments. ns, not significant. Bars, 10 µM.

To understand whether different cell types have the same ability to cluster supernumerary centrosomes, we induced centrosome amplification in a panel of six nontransformed cell lines: MCF10A (human mammary epithelium), HaCaT (human keratinocytes), J3B1A (human mammary epithelium), RPE-1 (human retinal pigment epithelium), NIH-3T3 (mouse fibroblasts), and BJ (human fibroblasts). Centrosome amplification was induced with dihydrocytochalasin B (DCB), an actin-depolymerizing drug that induces cytokinesis failure and tetraploidy. Strikingly, when scoring the percentage of bipolar divisions with extra centrosomes (>4 centrioles), we found that at cytokinesis we could distinguish two categories: cell lines that do not cluster efficiently (∼40% clustering efficiency) and cell lines that reached ∼80% of clustering efficiency (Fig. 1 A). At metaphase, this trend was less clear, presumably because of the presence of a mixed cell population at this stage, in which some cells have already clustered supernumerary centrosomes and others remain in a multipolar configuration (Fig. S1 A). Live-cell imaging confirmed that in cells that cluster, clustering occurs before anaphase onset (Fig. 1 B; and Videos 1 and 2). The clustering ability of the different cell lines was also validated by live-cell imaging analyses of cells expressing histone H2B-GFP after DCB treatment (Fig. 1, C and D). Thus, quantification of centrosome clustering after anaphase onset is overall more representative of the outcome of cell division. Differences in clustering efficiency were further confirmed using two additional methods to induce supernumerary centrosomes; the myosin II inhibitor blebbistatin, which also prevents cytokinesis, and cells treated with the CDK1 inhibitor RO-3306, which induces centriole overduplication in G2-arrested cells (Fig. S1, B–D; Loncarek et al., 2010). We found that these differences were not caused by centrosome inactivation, characterized by low centrosomal levels of pericentrin and γ-tubulin, as previously observed in *Drosophila* (Basto et al., 2008; Sabino et al., 2015; Fig. S1, F and G). To determine whether changes in clustering efficiency were the result of time spent in mitosis, we treated cells that do not cluster effectively, such as MCF10A and HaCaT cell lines, with the proteasome inhibitor MG132 to halt cells in metaphase (Basto et al., 2008; Kwon et al., 2008; Yang et al., 2008). After 4 h of treatment, MG132 was washed out, and cells were able to progress through mitosis (Fig. 1 E). Under these conditions, the clustering efficiency of MCF10A and HaCaT cells did not improve, suggesting that poor clustering in epithelial cells cannot be overcome by extending time in mitosis (Fig. 1 F). In contrast, MG132 treatment in RPE-1 and NIH-3T3 significantly improved clustering in metaphase, particularly in NIH-3T3 cells (Fig. S1 E), suggesting that although time in metaphase can improve clustering efficiency, this is not the case for all cell types. Our data demonstrate that different cell lines have varied abilities to cluster extra centrosomes.

### E-cadherin prevents efficient centrosome clustering in nontransformed cell lines

**Figure 2.**
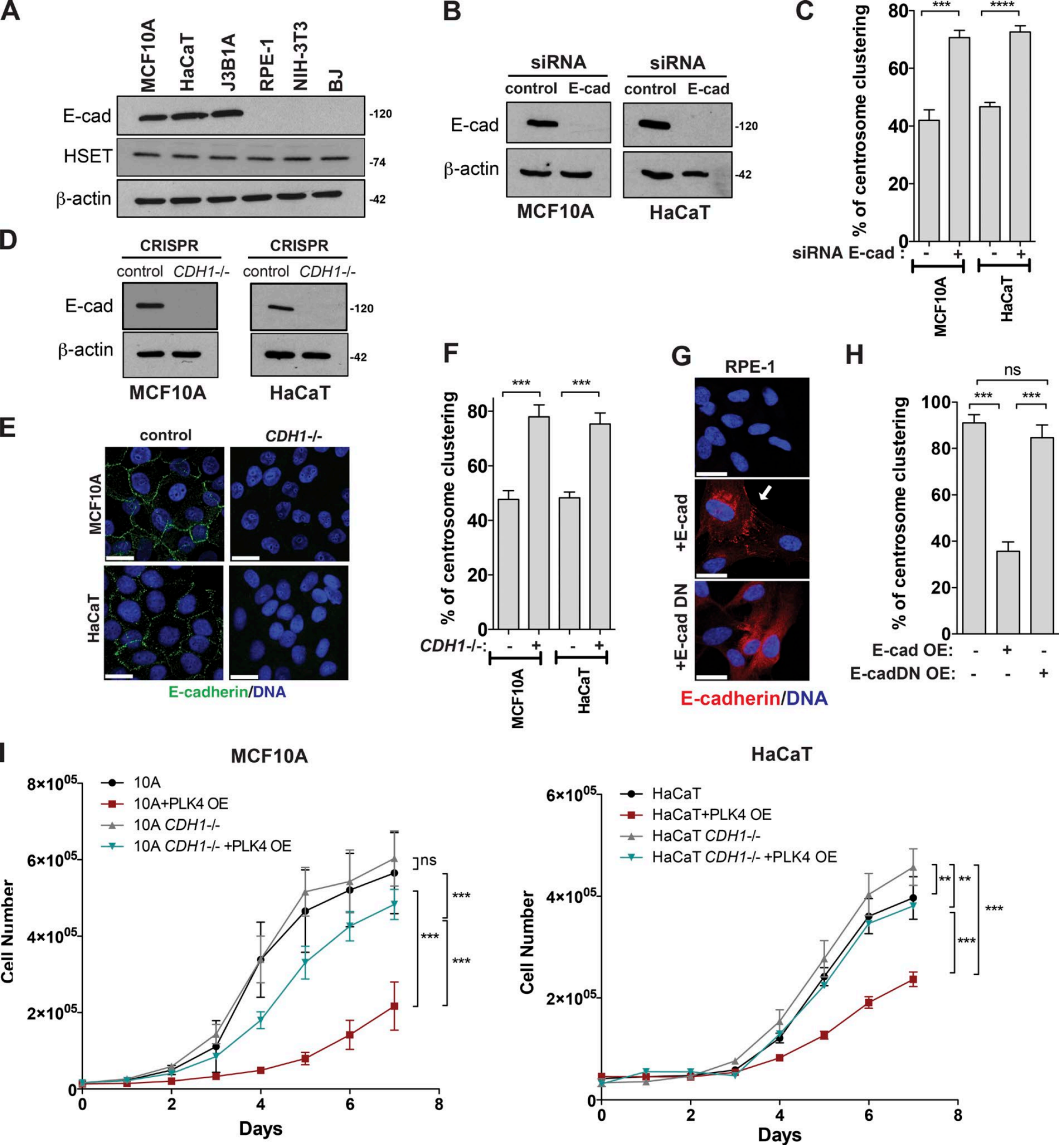
**Loss of E-cadherin promotes efficient centrosome clustering in nontransformed cell lines.** (A) Western blot analysis of E-cadherin and HSET levels in a panel of nontransformed cell lines. (B) Western blot analysis of E-cadherin levels in MCF10A and HaCaT cells after siRNA depletion of E-cadherin. (C) Quantification of centrosome clustering in cytokinesis upon DCB treatment in E-cadherin depleted cells (*n* = 150). (D) Western blot analysis of E-cadherin levels in MCF10A and HaCaT cells upon CRISPR-Cas9 knockout of E-cadherin (*CDH1*^−/−^; five knockout clones combined for each cell line). (E) Immunofluorescence images of control and *CDH1*^−/−^ MCF10A and HaCaT cells stained for E-cadherin (green) and DNA (blue). (F) Quantification of centrosome clustering in cytokinesis in control and *CDH1*^−/−^ cells (*n* = 150). (G) Immunofluorescence images in RPE-1 cells expressing WT E-cadherin and E-cadherin DN. Cells were stained for E-cadherin (red) and DNA (blue). White arrow highlights the cell–cell junctions. (H) Quantification of centrosome clustering in cytokinesis in RPE-1 cells expressing E-cadherin and E-cadherin DN (*n* = 150). (I) Analyses of the survival curves in control and *CDH1*^−/−^ MCF10A and HaCaT cells upon induction of centrosome amplification via PLK4 overexpression (PLK4 OE). For all graphics, error bars represent mean ± SD from three independent experiments. **, P < 0.01; ***, P < 0.001; ****, P < 0.0001; ns, not significant. Bars, 20 µM.

We found that the levels and localization of HSET, the major regulator of centrosome clustering, do not correlate with better clustering (Figs. 2 A and S1 H). However, a noticeable difference between cell lines that do not cluster efficiently, namely MCF10A, HaCaT, and J3B1A, is that they are of epithelial origin and thus express E-cadherin (Fig. 2 A). This is in contrast to RPE-1 cells, which are thought to also be of epithelial origin but do not express E-cadherin, and the NIH-3T3 and BJ fibroblasts (Fig. 2 A). To test if E-cadherin expression compromises clustering efficiency, we depleted E-cadherin by siRNA in MCF10A and HaCaT cells and found that this was sufficient to induce centrosome clustering to a level comparable to that of nonepithelial cells (Fig. 2, B and C). To confirm these results, we generated E-cadherin (*CDH1* gene) CRISPR-Cas9 knockout MCF10A and HaCaT cell lines. Analyses of individual clones (Fig. S2, A–F) or combined clones (Fig. 2, D–F) showed that *CDH1^–^*^/–^ cells efficiently clustered extra centrosomes, similarly to RPE-1, NIH-3T3, and BJ cells. Consistent with previous literature, loss of E-cadherin is not sufficient to induce epithelial-to-mesenchymal transition (EMT) in these cells, as assessed by expression of N-cadherin and vimentin (Fig. S2, C and F; Chen et al., 2014). Furthermore, adhesion molecules such as β-catenin and p120 catenin still localize to the sites of cell–cell contacts in *CDH1^–^*^/–^ cells (Fig. S2, G and H). Although we cannot exclude that the mislocalization of other cell–cell adhesion molecules could contribute to this process, our data suggest that it is the loss of E-cadherin itself, and not changes associated with EMT or loss of p120/β-catenin, that promotes efficient clustering. Increased clustering ability was also observed in the *CDH1^–^*^/–^ cells upon induction of supernumerary centrosomes by transient overexpression of Polo-like kinase 4 (PLK4) using a Tet-inducible system (Fig. S2, I–K; Godinho et al., 2014). Note that that overexpression of the TetR alone (that prevents PLK4 overexpression in the absence of doxycycline) partially increased clustering efficiency in epithelial cells upon DCB treatment (Fig. S2 K), suggesting that TetR overexpression is unexpectedly affecting this process. Conversely, overexpression of full-length E-cadherin in RPE-1 cells, but not an E-cadherin truncated mutant that lacks extracellular domains (E-cad DN) and does not form AJs, prevents efficient centrosome clustering in RPE-1 cells (Fig. 2, G and H). Consequently, E-cadherin knockout rescued the loss of viability observed in epithelial cells with extra centrosomes (Fig. 2 I). Altogether, these results demonstrate that E-cadherin expression prevents efficient centrosome clustering and that E-cadherin loss could be necessary to allow the survival of cancer cells with multiple centrosomes.

### Inhibition of cortical contractility prevents efficient centrosome clustering in cells lacking E-cadherin

**Figure 3.**
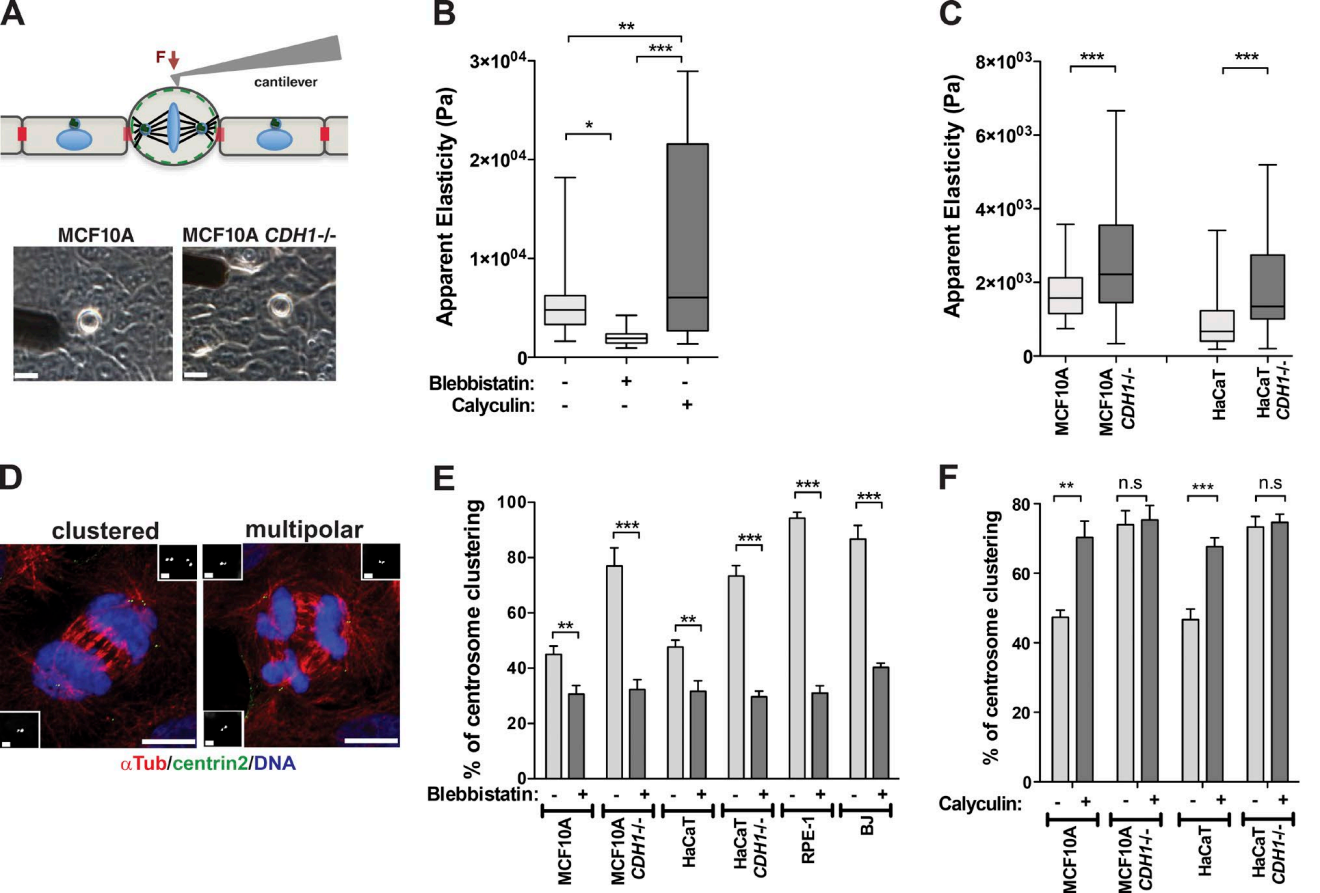
**Cortical contractility facilitates centrosome clustering in cells that do not express E-cadherin.** (A, top) Schematic representation of AFM experiment. (Bottom) Bright-field images of chosen metaphase cells used for the stiffness measurements. Cantilever can also be observed in these images. Bar, 20 µM. (B) Quantification of apparent elasticity (Pa) in metaphase cells treated with blebbistatin (50 µM, 4 h) and calyculin A (1 µM, 2 h). (C) Quantification of apparent elasticity (Pa) in metaphase cells within a monolayer. (D) Immunofluorescence images depicting examples of bipolar clustered and multipolar telophases. Cells were stained for microtubules (α-Tub, red), centrioles (centrin2, green), and DNA (blue). Bar, 10 µM. (Inset) High magnification of centrioles. Bar, 1 µM. (E) Quantification of centrosome clustering in telophase upon blebbistatin treatment (50 µM, 4 h; *n* = 150). (F) Quantification of centrosome clustering in cytokinesis upon treatment with calyculin A (1 µM, 2 h; *n* = 150). For all graphics, error bars represent mean ± SD from three independent experiments. *, P < 0.05; **, P < 0.01; ***, P < 0.001; ns, not significant.

In epithelial cells, down-regulation of cortical contractility at the sites of cell–cell contacts is achieved via inhibition of the RhoA-ROCK pathway downstream of E-cadherin (Hidalgo-Carcedo et al., 2011). We hypothesized that E-cadherin, which is still localized to the sites of cell–cell adhesion during mitosis (Baker and Garrod, 1993; den Elzen et al., 2009), prevents efficient clustering by decreasing cortical contractility in epithelial cells. To test this idea, we used atomic force microscopy (AFM) to measure mitotic cell apparent elasticity (Fig. 3 A; Harris and Charras, 2011). Apparent elasticity is dependent on tension generated by actomyosin contractility and thus can be used as a surrogate of cortical contractility (Harris et al., 2014). Indeed, inhibition of cortical contractility with blebbistatin decreases elasticity, whereas increasing contractility with calyculin A leads to an increase in cortical elasticity in mitotic MCF10A cells (Fig. 3 B). We found that *CDH1^–^*^/–^ cells had increased apparent elasticity compared with control cells expressing E-cadherin (Fig. 3 C). To investigate whether efficient centrosome clustering requires cortical contractility, we treated cells with blebbistatin to decrease actomyosin contractility. Because blebbistatin prevents ingression of the cleavage furrow and blocks cytokinesis, we quantified centrosome clustering at telophase (Fig. 3 D). Inhibition of cortical contractility dramatically prevented efficient clustering in cells that do not express E-cadherin, leading to a basal level of centrosome clustering of ∼30% in all cell lines (Fig. 3 E). Increasing myosin II activity and cortical contractility using the phosphatase inhibitor calyculin A enhanced centrosome clustering to ∼70% in cells expressing E-cadherin; however, it did not further improve clustering in *CDH1^–^*^/–^ cells (Fig. 3 F). Collectively, these results suggest that the presence of E-cadherin at cell–cell adhesion sites negatively regulates cortical contractility during mitosis, preventing centrosome clustering.

### Cortical localization of DDR1 downstream of E-cadherin prevents efficient centrosome clustering via RhoE

**Figure 4.**
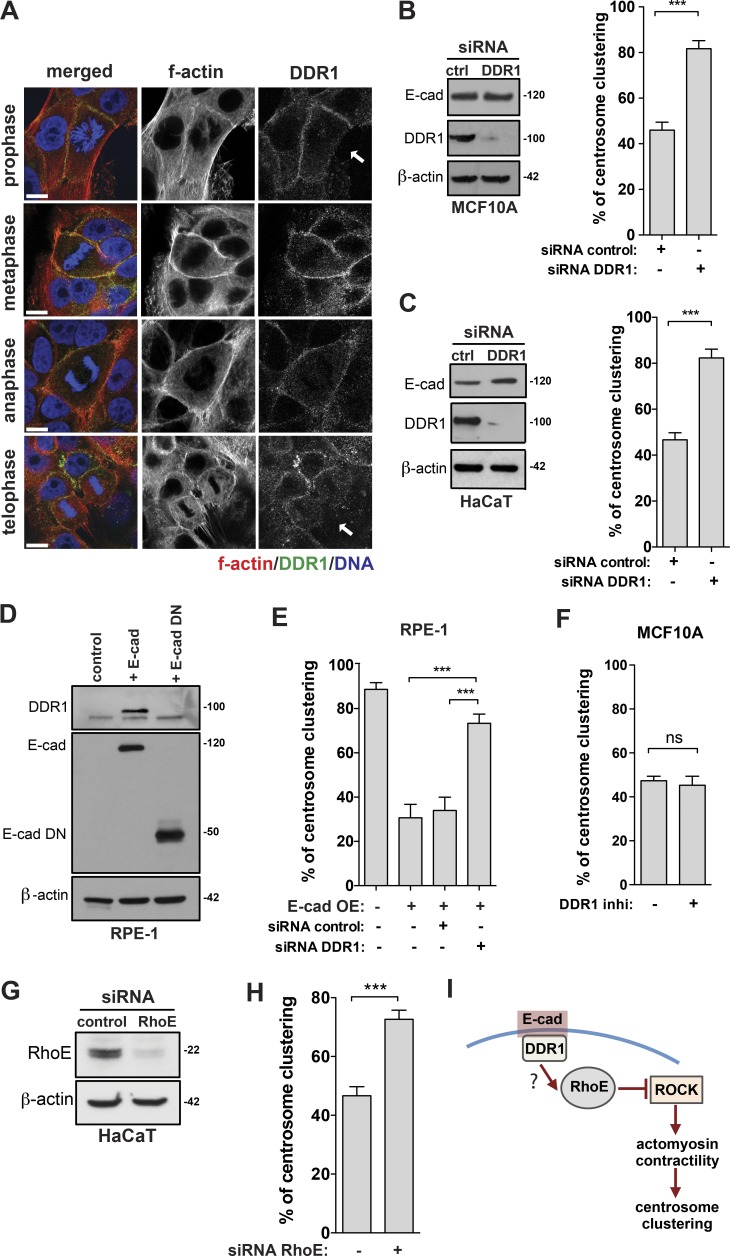
**Cortical localization of DDR1 in cells expressing E-cadherin prevents efficient centrosome clustering.** (A) Immunofluorescence images showing cortical localization of DDR1 during mitosis. Cells were stained for F-actin (phalloidin, red), DDR1 (green), and DNA (blue). White arrows represent areas where there are no cell–cell contacts. (B, left) Western blot analysis of DDR1 and E-cadherin levels after siRNA depletion of DDR1 in MCF10A cells. (Right) Quantification of centrosome clustering in cytokinesis upon DDR1 depletion. (C, left) Western blot analysis of DDR1 and E-cadherin levels after siRNA depletion DDR1 in HaCaT cells. (Right) Quantification of centrosome clustering in cytokinesis upon DDR1 depletion. (D) Western blot analysis of the levels of E-cadherin and DDR1 in RPE-1 cells expressing exogenous WT E-cadherin and E-cadherin DN. (E) Quantification of centrosome clustering in cytokinesis in RPE-1 cells expressing E-cadherin and E-cadherin DN before and after DDR1 depletion by siRNA (*n* = 150). (F) Quantification of centrosome clustering in metaphase and cytokinesis in MCF10A cells treated with 15 µM DDR1 inhibitor for 3 h. (G) Western blot analysis of RhoE levels in HaCaT cells after siRNA depletion of RhoE. (H) Quantification of centrosome clustering in cytokinesis upon RhoE depletion (*n* = 150). (I) Schematic representation of RhoE-mediated regulation of cortical contractility downstream of E-cadherin. For all graphics, error bars represent mean ± SD from three independent experiments. ***, P < 0.001; ns, not significant. Bar, 10 µM.

Recruitment of DDR1 to the AJs, which requires E-cadherin, plays important roles in reducing cortical contractility (Hidalgo-Carcedo et al., 2011). To test whether DDR1 was preventing efficient clustering in epithelial cells, we first assessed DDR1 localization during mitosis. We found that DDR1 localized to the cortex in epithelial cells during mitosis; however, this localization was absent in areas where there were no cell–cell contacts (Fig. 4 A, arrows). DDR1 mitotic localization was not maintained in cells deficient in E-cadherin expression, because DDR1 protein levels were dramatically reduced upon E-cadherin knockdown (Fig. S3 A). This is likely caused by a decrease in the stability or translation of DDR1, since loss of E-cadherin does not affect DDR1 mRNA expression (Fig. S3 B). This is consistent with the fact that DDR1 is mainly expressed in epithelial cells, which was also confirmed in our panel of nontransformed cell lines (Fig. S3 C; Leitinger, 2014). DDR1 depletion by siRNA in epithelial cells led to efficient centrosome clustering without affecting E-cadherin protein levels or localization (Fig. 4, B and C; and Fig. S3 D). This suggests that DDR1 is downstream of E-cadherin. Overexpression of E-cadherin in RPE-1 cells, but not of E-cad DN, is sufficient to induce DDR1 protein stabilization (Fig. 4 D), further supporting a role for E-cadherin in DDR1 regulation. DDR1 depletion in RPE-1 cells overexpressing E-cadherin also improved centrosome clustering (Fig. 4 E). Moreover, the regulation of DDR1 levels and centrosome clustering by E-cadherin is maintained in cancer cells, as demonstrated by the CRISPR-Cas9 knockout of E-cadherin in the human squamous carcinoma line A431 (Fig. S4, A–E). Regulation of cortical contractility by DDR1 during interphase is independent of its tyrosine kinase activity but mediated via the recruitment of p190RhoGAP, which inhibits RhoA activity (Hidalgo-Carcedo et al., 2011). Consistently, we found that chemical inhibition of DDR1 kinase activity had no effect on the clustering of extra centrosomes (Fig. 4 F). However, depletion of p190RhoGAP did not increase the clustering ability of epithelial cells (Fig. S5, A and B). Similar results were obtained upon depletion of DLC3, another negative regulator of RhoA at the AJs (Fig. S5, C and D; Hendrick et al., 2016). It is known that the small GTPase RhoE, which is recruited to the AJs in a DDR1-dependent manner, can also negatively regulate actomyosin contractility by directly inhibiting ROCK I activity (Riento et al., 2003; Hidalgo-Carcedo et al., 2011). Indeed, depletion of RhoE by siRNA in epithelial cells improves centrosome clustering (Fig. 4, G and H). Collectively, these results suggest that down-regulation of actomyosin contractility via DDR1 and RhoE prevents efficient clustering in epithelial cells (Fig. 4 I).

### Cortical contractility facilitates HSET-mediated centrosome clustering

**Figure 5.**
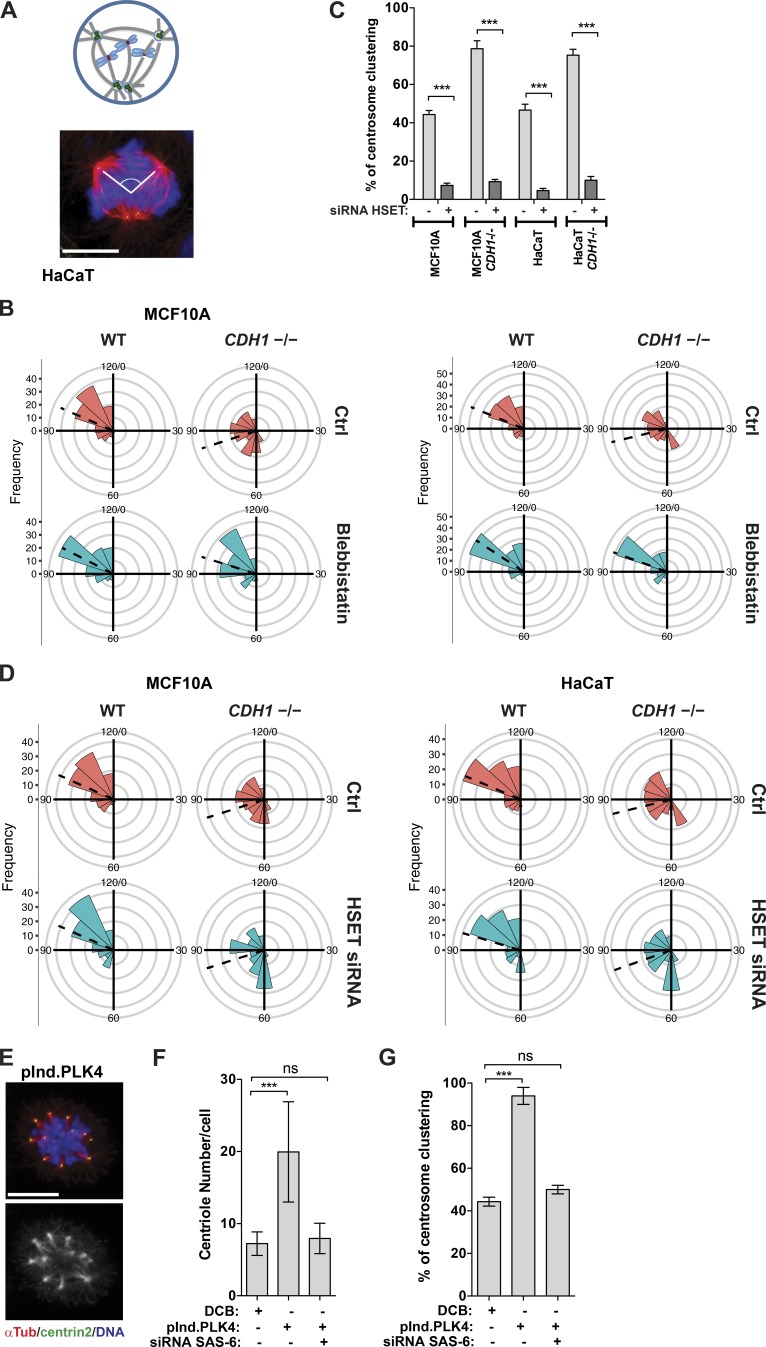
**Inhibition of cortical contractility downstream of E-cadherin/DDR1 complex inhibits centrosome movement.** (A) Representation of the angles measured between the two closest poles in tripolar metaphases. Cells were stained for microtubules (α-Tub, red), centrioles (centrin2, green), and DNA (blue). Bar, 10 µm. (B) Rose plot showing the frequency of the angles measured in MCF10A and HaCaT cells (control and *CDH1*^−/−^) upon blebbistatin treatment (50 µM, 4 h; *n* = 150). Dashed line represents the mean angle distribution. (C) Quantification of centrosome clustering in cytokinesis upon depletion of HSET by siRNA (48 h). (D) Rose plot showing the frequency of the angles measured in MCF10A and HaCaT cells (control and *CDH1*^−/−^) upon HSET siRNA. Dashed line represents the mean angle distribution. (E) Immunofluorescence images of mitotic cells with high levels of extra centrosomes. Cells were stained for microtubules (α-Tub, red), centrioles (centrin2, green), and DNA (blue). Bar, 10 µm. (F) Quantification of number of centrioles per mitotic cell in cells overexpressing PLK4 treated or not with SAS-6 siRNA for 48 h (*n* = 150). (G) Quantification centrosome clustering in cells overexpressing PLK4 treated or not with SAS-6 siRNA for 48 h. For all graphics, error bars represent mean ± SD from three independent experiments. ***, P < 0.001; ns, not significant.

Cortical actomyosin contractility was shown to drive the movement of the microtubule asters during early mitosis (Rosenblatt et al., 2004). We hypothesized that the proximity of these asters was necessary to bring centrosomes together to be clustered by HSET. To test this idea, we first measured the smallest angle between centrosomes in tripolar metaphases in epithelial cells with or without E-cadherin (Fig. 5 A). We found that the distribution of the smallest angle varies, with the *CDH1^–^*^/–^ cell lines displaying on average smaller angles, suggesting that centrosomes are closer (Fig. 5 B). Inhibition of cortical contractility with blebbistatin abolished this effect, leading to similar angle distribution between control and *CDH1^–^*^/–^ cells (Fig. 5 B). This was further confirmed using a ROCK I inhibitor (Fig. S5 E). Conversely, increasing myosin II activity with calyculin A leads to a smaller angle distribution in epithelial cells (Fig. S5 F). Cortical forces are transmitted to the centrosomes via astral microtubules, which have been previously implicated in centrosome clustering (Kwon et al., 2008). Depletion of astral microtubules with low doses of nocodazole also leads to clustering defects and an increase in the smallest angle distribution in tripolar metaphases in the *CDH1^–^*^/–^ cell lines (Fig. S5, G and H). We propose that the proximity of centrosomes in *CDH1^–^*^/–^ cell lines is essential to allow them to be clustered by the minus end–directed microtubule motor HSET (Fig. S6 A). This model predicts that although HSET is essential for clustering, its depletion should not affect centrosome proximity. Depletion of HSET prevented centrosome clustering in all cell lines independently of E-cadherin expression, leading to basal levels of clustering <10%, the lowest we observed (Figs. 5 C and S6 B). However, unlike inhibition of cortical contractility, HSET depletion does not affect the smallest angle distribution in tripolar metaphases (Fig. 5 D). This suggests that centrosomes can still be close to each other but, because of HSET absence, they cannot cluster. This could also explain why HSET expression is not sufficient to promote efficient clustering in all cell lines (Fig. 2 A). Our model foresees that there is a minimal distance required between extra centrosomes for HSET to exert its function. To test this idea, we took advantage of a newly generated construct to transiently overexpress PLK4 (pInducer.PLK4; see Materials and Methods for details), which led to the generation of a larger number of supernumerary centrosomes per cell that are consequently more likely to be closer in proximity to one another. Addition of doxycycline led to a mean of ∼20 centrioles per mitotic cell, compared with ∼8 centrioles induced by DCB treatment or our previous PLK4 construct (Fig. 5, E and F; and Fig. S2 L). Remarkably, this was sufficient to allow efficient clustering of supernumerary centrosomes in epithelial cells expressing E-cadherin (∼100%) compared with DCB-treated cells (Fig. 5 G). Decreasing centrosome numbers by depletion of the centrosomal protein SAS-6 in cells overexpressing PLK4 prevents efficient clustering (Fig. 5 G). Collectively, these results suggest that cortical contractility in cells without E-cadherin facilitates centrosome clustering by enabling the close proximity of extra centrosomes, a process required for HSET to cluster centrosomes via its minus-end motor activity and its ability to cross-link and slide microtubules (Cai et al., 2009).

### Centrosome tracking reveals a biphasic clustering mechanism

**Figure 6.**
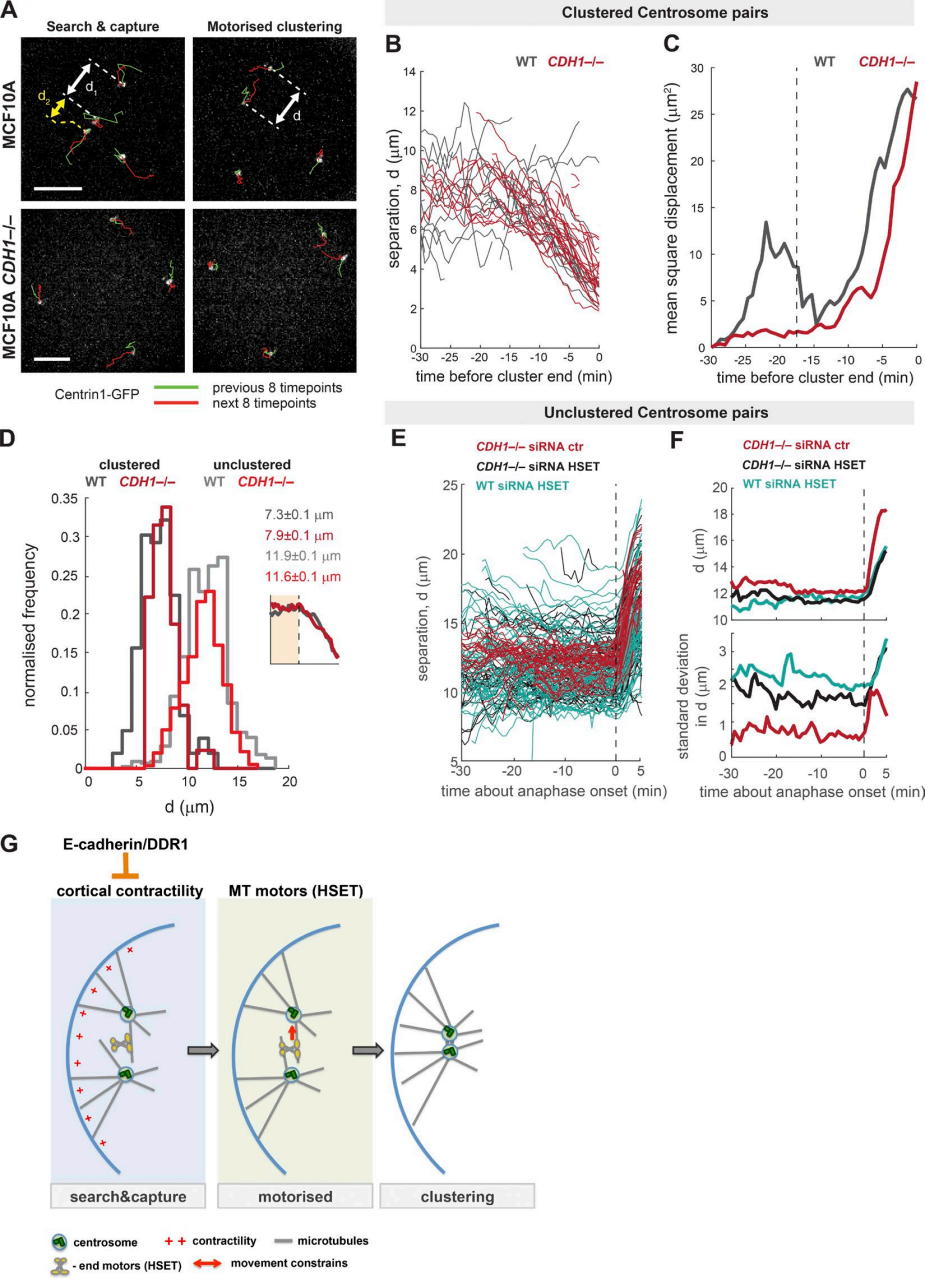
**Cortical contractility restricts centrosome movement to promote HSET-mediated centrosome clustering.** (A) Representation of live 3D measurements of distances between pairs of clustering centrosomes in MCF10A and MCF10A *CDH1*^−/−^ cells. Green and red lines represent the centrosomes’ trajectories during the eight time points (every 40 s) before and after the image shown, respectively. *d1* and *d2* represent centrosome distance. (B) Graphic depicting centrosome distance, *d*, over time for each successfully clustering pair (WT, *n* = 31; *CDH1*^−/−^, *n* = 40). Cluster completion was defined as the time point at which separation stabilized. (C) Population mean square displacement of centrosome separation during centrosome clustering (WT, *n* = 31; *CDH1*^−/−^, *n* = 40). The vertical dashed line represents the transition between search-and-capture and motorized clustering phases. (D) Histograms of centrosome separation, *d*, during the search-and-capture phase of centrosome clustering, as seen in the schematic. Data are for centrosome pairs that successfully cluster (WT, *n* = 155; *CDH1*^−/−^, *n* = 102), and centrosome pairs that fail to cluster in cells that also contain a successful cluster event (WT, *n* = 683; *CDH1*^−/−^, *n* = 718). Values given are medians ± SEM. (E) Centrosome distance for each pair that fail to cluster (*CDH1*^−/−^ siRNA ctr, *n* = 70; *CDH1*^−/−^ siRNA HSET, *n* = 115; WT siRNA HSET, *n* = 141). Trajectories are aligned at anaphase onset. (F) The population mean (top) and SD (bottom) of nonclustered centrosome pairs in F, demonstrating the increased variability (SD) in *d* after loss of HSET, and further with inhibited cortical contractility. The vertical dashed line represents the anaphase B onset. (G) Schematic representation explaining the biphasic model for centrosome clustering. Bars, 10 µM.

To determine the minimal distance required for HSET to cluster extra centrosomes, we performed live-cell imaging of MCF10A and MCF10A *CDH1^–^*^/–^ cells expressing centrin1-GFP after DCB treatment (four centrosomes during mitosis). Software to track centrosome positioning in mitosis was used, and all events in which a pair of centrosomes managed to cluster were analyzed. First, we observed that centrosome clustering in MCF10A and MCF10A *CDH1^–^*^/–^ cells occurred just before anaphase onset in 87.5% and 81.8% of cases, respectively. This is consistent with our observations that by anaphase, most cells had clustered their extra centrosomes. Furthermore, analyses of centrosome movement during clustering revealed a biphasic clustering mechanism (Fig. 6, A and B; Fig. S6 H; and Videos 3 and 4). In the first phase, the centrosomes move slowly toward and away from each other over time, a process we termed the “search-and-capture” phase (Fig. 6 A, left column). In the second phase, centrosomes undergo continuous directed motion toward each other, which we propose to be a consequence of HSET-mediated clustering, or the “motorized” phase (Fig. 6 A, right column). Both control and *CDH1^–^*^/–^ cells present this biphasic movement of the centrosomes, and the time that centrosomes take to cluster during the motorized phase is invariably 15 min (Figs. 6 C and S6 H; beginning of the motorized phase is marked by a vertical dashed line). It is also clear that the distance at which centrosomes initiate the motorized phase of clustering is 7–8 µm, which we predict to be the distance required for HSET to bind to microtubules emanating from adjacent centrosomes (Fig. 6 D). By contrast, pairs of centrosomes that do not cluster showed a mean distance of ∼11–12 µm (Fig. 6 D). However, this minimal distance is not the only requirement for efficient clustering, as not all centrosomes that are close together will cluster (Fig. 6 A, yellow arrow). Our data showed that there is a significant difference between the range of motion in control and *CDH1^–^*^/–^ cells in the search-and-capture phase, where an increase in the random movement, given by the SD, of centrosomes toward and away from each other is observed compared with the knockout cells (Fig. S6 H). This suggests that increased cortical contractility in *CDH1^–^*^/–^ cells restricts centrosome movement, preventing centrosomes from moving away from each other, facilitating clustering. Consistently, the mean square displacement of the centrosomes is higher in control cells during the search-and-capture phase, where there are less cortical forces to constrain movement (Fig. 6 C). Once the centrosomes engage the motorized phase, the velocity at which they cluster does not change in control and *CDH1^–^*^/–^ cells, suggesting that the motorized clustering is unaffected by cortical contractility (Fig. S6 I). Our data demonstrate that it is not only the distance between centrosomes that is important, but the time centrosomes spend nearby one another also increases the probability that they establish stable interactions necessary for HSET binding, and therefore clustering. Depletion of HSET by siRNA confirmed our data, showing that cells lose the ability to cluster extra centrosomes (Fig. 5 C and Videos 5 and 6), and therefore no motorized phase was observed where centrosomes move toward each other (Fig. 6, B and C). As a consequence, centrosomes lose the biphasic movement, and their separation changes only at anaphase B onset, when the spindle elongates (Fig. 6, E and F). Interestingly, although loss of HSET does not affect the SD of centrosome movement in control cells (∼2 µm; Figs. 6 F and S6 H), HSET depletion increases the SD in *CDH1^–^*^/–^ cells (from 1 to 2 µm, similar to control cells; Fig. 6 F). This suggests that loss of HSET suppresses the movement constraints imposed by cortical contractility. We propose a model wherein increased cortical contractility in cells that do not express E-cadherin restricts stochastic centrosome movement in the presence of HSET, which is then required for HSET to stably bind to microtubules to promote centrosome clustering (Fig. 6 G).

### Loss of E-cadherin and DDR1 correlates with high levels of centrosome amplification in breast cancer

**Figure 7.**
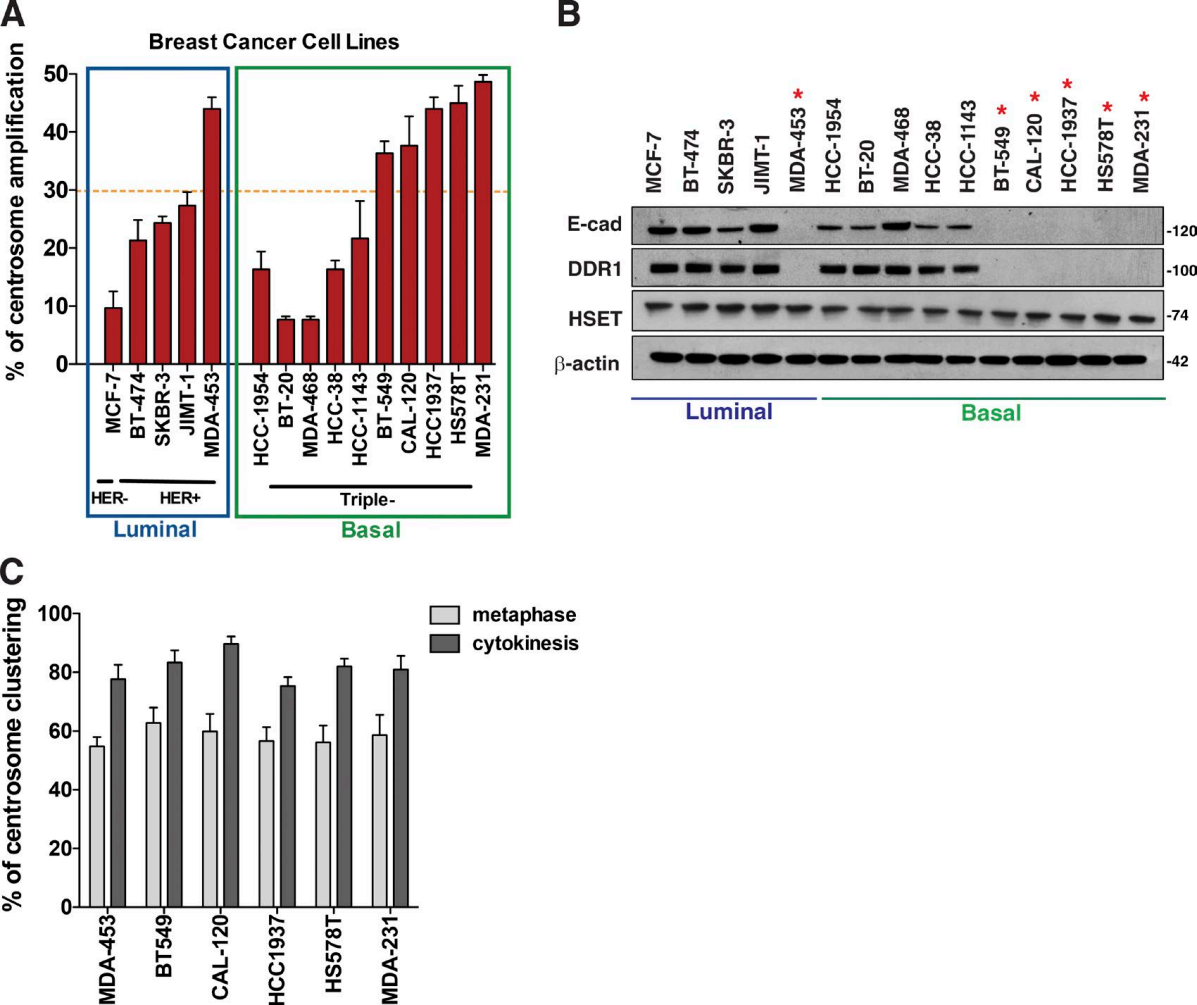
**Loss of E-cadherin and DDR1 correlates with high levels of centrosome amplification in breast cancer.** (A) Quantification of centrosome numbers in a panel of breast cancer cell lines. (B) Western blot analysis of E-cadherin, DDR1, and HSET expression in breast cancer cell lines. Red asterisk marks the cell lines with high levels of centrosome amplification. (C) Quantification of centrosome clustering in metaphase and cytokinesis in cells with high levels of centrosome amplification. Error bars represent mean ± SD from three independent experiments.

Most solid tumors, which are of epithelial origin, have some degree of centrosome amplification (Zyss and Gergely, 2009; Chan, 2011). However, our data suggest that epithelial cells (nontransformed and transformed) have low probability of proliferating and surviving in the presence of extra centrosomes because of inefficient clustering mechanisms. Thus, it is possible that these cells might have mechanisms to cope with the presence of supernumerary centrosomes, and loss of E-cadherin or DDR1 could be part of this adaptation mechanism. To assess this, we analyzed a panel of 15 breast cancer cell lines for centrosome amplification and E-cadherin/DDR1 levels. We found that the six cell lines with a high fraction of cells carrying extra centrosomes (>30%) invariably lost E-cadherin protein expression, and this was independent of breast cancer subtype (Fig. 7, A and B), although higher levels of centrosome amplification are associated with basal cell lines, as previously shown (D’Assoro et al., 2002; Denu et al., 2016). In agreement with our data showing that the presence of E-cadherin is important for DDR1 stabilization, DDR1 protein is lost in the same cell lines. Furthermore, the levels of HSET do not correlate with centrosome amplification in the cell lines analyzed (Fig. 7 B). As predicted, the six cell lines with high levels of centrosome amplification and no E-cadherin expression cluster extra centrosomes very efficiently (∼80%; Fig. 7 C). These results suggest that loss of E-cadherin could allow epithelial tumors to tolerate centrosome amplification.

## Discussion

Cells have intrinsic mechanisms that facilitate centrosome clustering (Godinho and Pellman, 2014). Thus, it is thought that cells are unlikely to require adaptation to centrosome amplification, which is further supported by the fact that most cancer cell lines with extra centrosomes are able to cluster centrosomes efficiently (Ring et al., 1982; Quintyne et al., 2005; Kwon et al., 2008; Ganem et al., 2009). However, our findings challenge this idea and indicate that at least in epithelial tumors, cancer cells need to adapt to efficiently proliferate in the presence of supernumerary centrosomes. We demonstrate that induction of centrosome amplification in a panel of nontransformed cell lines reveals intrinsic differences in clustering ability, with epithelial cells displaying an inefficient process. These differences are not caused by centrosome inactivation, as previously shown in *Drosophila* cells with extra centrosomes (Sabino et al., 2015), highlighting that the prevalence of mechanisms that allow the formation of pseudo-bipolar spindles and survival of cells with supernumerary centrosomes varies between cell types and organisms.

Our results show that the presence of E-cadherin in epithelial cells affects the cortical properties of cells in mitosis, particularly contractility, likely through DDR1. DDR1 recruitment to the AJs during interphase was shown to decrease actomyosin contractility through a signaling cascade involving the RhoA negative regulator p190RhoGAP (Hidalgo-Carcedo et al., 2011). Here we showed that DDR1 localizes to the cortex in epithelial cells during mitosis and that its expression prevents efficient centrosome clustering. However, in this context, DDR1 regulation of cortical contractility is not mediated by p190RhoGAP but by RhoE, which can directly inhibit ROCK I (Riento et al., 2003). Our data show that although E-cadherin is important for DDR1 stabilization or translation, loss of DDR1 does not affect E-cadherin levels, similar to what has been observed in the MDCK epithelial cell line (Eswaramoorthy et al., 2010), or the localization of the cell–cell adhesion molecules β-catenin and p120. Thus we conclude that the regulation of contractility by DDR1, and not AJs, per se, plays a role in centrosome clustering.

Centrosome tracking showed that centrosome movement during clustering occurs in a biphasic manner. The first step is a search-and-capture phase characterized by slow movement of centrosomes, and the second step is a motorized phase, in which centrosomes engage in fast directional movement. HSET depletion abolishes the motorized phase, suggesting that it is HSET mediated. Interestingly, although the motorized phase of centrosome clustering remains similar, the search-and-capture phase is strongly affected by the presence of E-cadherin. We found that the presence of E-cadherin leads to larger centrosome displacement in the early stages of clustering, suggesting that its loss restricts centrosome movement, a restriction that is HSET dependent. We propose that the restriction of centrosome movement in the search-and-capture phase is mediated by cortical contractility, facilitating HSET binding to microtubules emanating from different centrosomes and promoting clustering during the motorized phase (Fig. 6 G). This model also explains why the presence of HSET itself is not sufficient to ensure efficient clustering of supernumerary centrosomes. Importantly, similar biphasic behavior has been observed for the rate of poleward chromosome movement during anaphase, where an initial slow rate precedes a faster movement that is also mediated by a microtubule motor, dynein (Sharp et al., 2000).

Our findings highlight the stochastic nature of centrosome clustering. There seems to be a random probability for centrosomes to be at the right distance to allow HSET to cluster centrosomes. This could help explain why ∼40% of epithelial cells successfully cluster supernumerary centrosomes. However, restriction of centrosome movement by cortical contractility increases the probability of these centrosomes establishing stable interactions, thereby increasing efficiency of clustering. How contractility restricts centrosome movement during mitosis remains unclear. One possibility is by regulating microtubule-pulling forces that are generated by motors at the cortex, such as dynein. It has been proposed previously that efficient pulling forces important for spindle positioning require the microtubule plus ends to be anchored to a relatively stiff cortex (Carreno et al., 2008; Kunda et al., 2008). Indeed, actomyosin contractility was shown to be important for dynein-mediated pulling forces on the microtubules and to prevent membrane invaginations at the sites of microtubule pulling forces in *Caenorhabditis elegans* embryos (Redemann et al., 2010; De Simone et al., 2016). Thus, it is possible that in epithelial cells, low contractility could lead to inefficient microtubule pulling forces at the cortex, leading to increased random centrosome movement that prevents efficient centrosome clustering.

As strategies emerging from basic biology to inhibit centrosome clustering provide the rationale for the development of specific inhibitors, stratification of patients for potential response to treatment with such compounds becomes essential (Rebacz et al., 2007; Kwon et al., 2008; Ganem et al., 2009; Karna et al., 2011; Watts et al., 2013; Wu et al., 2013). Quantification of centrosome number in tumors is highly time-consuming and cumbersome; therefore, clinical diagnosis would be better suited by the identification of an easily applicable biomarker to identify tumors containing extra centrosomes. Loss of E-cadherin–mediated adhesion, via epigenetic or genetic mechanisms, has been observed in many epithelial tumors and is often associated with higher tumor grade and poor prognosis (Birchmeier and Behrens, 1994). We propose that loss of E-cadherin, routinely assessed in clinical pathology by immunohistochemistry, may function as a biomarker for centrosome amplification. Indeed, we found a strong association between loss of E-cadherin and a high fraction of cells carrying extra centrosomes in breast cancer cell lines. Furthermore, in breast cancer, both loss of E-cadherin and centrosome amplification have been independently associated with poor prognosis and more aggressive tumors (e.g., triple-negative breast cancer; D’Assoro et al., 2002; Kashiwagi et al., 2010; Denu et al., 2016). Although it is unclear whether loss of E-cadherin is a prerequisite for the maintenance of extra centrosomes or whether centrosome amplification itself requires adaptation, these observations suggest that centrosome amplification and loss of E-cadherin might coevolve during tumor progression.

## Materials and methods

### Cell culture

Cell lines were maintained at 37°C with 5% CO_2_ atmosphere. Specific growth medium can be found in Table S1. Breast cancer cell lines were a gift from P. Schmidt (Barts Cancer Institute-Queen Mary University of London, London, England, UK). Tetracycline-free FBS (Hyclone) was used to grow cells expressing the *PLK4* Tet-inducible construct, with the exception of MCF10A cells, for which horse serum was always used.

### Lentiviral vectors

To generate cell lines overexpressing PLK4, we used the lentiviral vectors pLenti-CMV-TetR-Blast (17492; Addgene) and p-Lenti-CMV/TO-Neo-Dest (17292; Addgene; Campeau et al., 2009; Godinho et al., 2014). PLK4 cDNA was cloned using the Gateway system into the pLenti-CMV/TO-Neo-Dest vector. Cell lines were initially infected with a lentivirus containing the TetR and selected using Blasticidin (5–10 µg/ml). After selection, cells were then secondarily infected with the PLK4-containing lentivirus and selected with Geneticin (100–200 µg/ml). The selected cells were maintained as a pool to make a cell population. To induce high levels of centrosome amplification per cell, we cloned the PLK4 cDNA using the Gateway system into the pInducer21 vector (46948; Addgene; Meerbrey et al., 2011). Lentilox Centrin1-eGFP construct was a gift from J. Loncarek (National Institutes of Health, Bethesda, MD). To overexpress E-cadherin, we used pWZL-blast-DN-E-cadherin (18800; Addgene) and pWZL-blast-E-cadherin (18804; Addgene; Onder et al., 2008). The LV-GFP plasmid (25999; Addgene) was used to express H2B-GFP (Beronja et al., 2010).

To generate lentivirus, HEK-293M were grown in antibiotic-free medium and cotransfected with the required lentiviral plasmid, VSV-G (pMD2.G 12259; Addgene) and Gag-Pol (psPAX2 12260; Addgene) using Lipofectamine 2000 (Invitrogen), according to the manufacturer’s instructions. Lentiviruses were harvested 24 and 48 h after infection, passed through a 0.45-µM syringe filter unit (Merck Millipore), and stored at −80°C. To infect cells, 8 µg/ml polybrene (Sigma-Aldrich) was added to 1.5 ml lentivirus and added on top of cells for 6 h. This process was repeated the next day, and 48 h after initial infection, cells were treated with appropriate antibiotic for selection or amplified for cell sorting.

### Chemicals

Doxycycline (Sigma-Aldrich) was used at 2 µg/ml. The following doses of inhibitors were used: 4 µM DCB (Sigma-Aldrich), 10 µM p38 inhibitor (SB203580; New England Biolabs), 50 µM blebbistatin (Sigma-Aldrich), 5 µM R0-3306 (CDK1i; Sigma-Aldrich), 1 µM calyculin A (Abcam), 15 µM DDR1-IN-1 (Tocris), 10 µM Y-27632 (ROCKi; Tocris), 10 µM MG132 (Tocris), and 5 nM nocodazole (Sigma-Aldrich).

### Indirect immunofluorescence

Cells plated on glass coverslips were washed in PBS and fixed with 4% formaldehyde for 15 min at room temperature. For centriole staining, cells were fixed with ice-cold methanol at −20°C for 10 min. For E-cadherin staining, cells were fixed with ice-cold 1:1 methanol/acetone at −20°C for 10 min. After fixation, cells were permeabilized in 0.2% Triton X-100 in PBS for 5 min and blocked in blocking buffer (PBS, 5% BSA, and 0.1% Triton X-100) for 30 min. Cells were then stained in primary antibodies diluted in blocking buffer for 60 min. Cells were washed with PBS and incubated with species-specific fluorescent secondary antibodies (Alexa Fluor conjugated; Molecular Probes). DNA was stained with Hoechst 33342 (1:5,000; Invitrogen) for 5 min in PBS. Antibodies used included anti–α-tubulin DM1α (1:1,000; Sigma-Aldrich), anti–centrin-2 N-17-R (1:100; Santa Cruz Biotechnology), anti–γ-tubulin GTU88 (1:500; Sigma-Aldrich), anti–E-cadherin HECD-1 (1:500; Abcam), anti–DDR1 1F10 and 7A9 (1:500; made by B. Leitinger [Carafoli et al., 2012]), anti-pericentrin (1:1,500; Abcam), anti-HSET (1:1,000; Bethyl Laboratories), anti–β-catenin (1:1,000; Abcam), and anti-p120 (1:1,000; BD Biosciences).

### Live-cell imaging

MCF10A, HaCaT, BJ, and RPE-1 cells expressing H2B-GFP were grown on glass-bottom dishes (MatTek) and treated with DCB for 18 h. Binucleated cells were imaged on an Olympus DeltaVision microscope (Applied Precision) equipped with a Coolsnap HQ camera. The microscope was enclosed within temperature and CO_2_-controlled environments that maintained an atmosphere at 37°C and 3–5% humidified CO_2_. GFP and bright-field images were captured at multiple points for 16 h at 40× (1.3 NA) objective. Captured images from each experiment were analyzed using the softWoRx Explorer software. Centrin1-GFP–expressing cells were plated onto 35-mm glass-bottom tissue culture dishes (Ibidi), treated with 4 µM DCB for 20 h, and washed out in complete medium for 24 h. Cells were imaged on an Eclipse Ti-E inverted microscope (Nikon) equipped with a CSU-X1 Zyla 4.2 camera (Ti-E, Zyla; Andor), including a Yokogawa Spinning Disk, a precision motorized stage, and Nikon Perfect Focus, all controlled by NIS-Elements Software (Nikon). The microscope was enclosed within temperature- and CO_2_-controlled environments that maintained an atmosphere of 37°C and 5% humidified CO_2_. Movies were acquired with a Plan Apochromat 100× 1.45-NA oil objective with a 0.13-mm working distance. Cells were imaged over 35 *z*-slices separated by 500 nm every 40 s until cell cytokinesis. Laser power in the 488-nm wavelength was set to 5%, with exposure time 50 ms per *z*-slice and 2 × 2 binning.

### Cell viability

H2B-GFP–expressing cells were plated in 12-well plates for 24 h to adhere. The plates were then placed in an IncuCyte Zoom (Essen Bioscience) for 7 d. GFP and bright-field images were taken every hour. Cell number was quantified using a mask for the number of GFP foci using IncuCyte Zoom software.

### Western blotting

Cells were collected and resuspended in RIPA buffer (Thermo Fisher Scientific) with added protease inhibitors (Roche). Protein concentration was quantified using the Bio-Rad DC protein assay (15 µg loaded per well). Protein samples were resuspended in Laemmli buffer and separated on SDS-PAGE and transferred onto PVDF membranes. Antibodies used included anti–β-actin 13E5 (1:5,000; Cell Signaling Technology), anti–E-cadherin HECD-1 (1:200; Abcam), anti–DDR1 C-20 (1:200; Santa Cruz Biotechnology), anti-KIFC1 (HSET; 1:500; Bethyl Laboratories), anti-Mad2 (1:500; Bethyl Laboratories), anti-RhoE (1:100; Sigma-Aldrich), anti-p190 (1:250; BD Biosciences), anti–STARD8 E-2 (DLC3; 1:100; Santa Cruz Biotechnology), anti–N-cadherin (1:500; BD Biosciences), anti–Vimentin RV202 (1:500; BD Biosciences), anti-ERM (1:500; Cell Signaling Technology), anti–pMLC T18/S19 (1:500; Cell Signaling Technology), and anti–pDDR1 Tyr513 (1:100; Origene). Western blots were developed using a SRX-101A Konica Minolta and scanned.

### siRNA

siRNA was performed using Lipofectamine RNAiMax (Invitrogen). 50 nM siRNA was used per well in a six-well plate. After 6-h incubation, transfected cells were washed, and normal growth medium was added. Cells were analyzed 48 h after transfection. siRNAs used were negative control (1027310; Qiagen), CDH1/E-cadherin (L-003877-00; Dharmacon), DDR1 (L-003111-00; Dharmacon), SAS-6 (M-004158-02; Dharmacon), p190RhoGAP (M-004158-02; Dharmacon), STARD8/DLC3 (M-010254-00; Dharmacon), KIFC1/HSET (L-004958-00; Dharmacon), and RND3/RhoE (J-007794-09; Dharmacon). Note: We noticed that siRNA against RhoE can cause a strong spindle checkpoint phenotype because of unspecific Mad2 depletion. Several siRNA sequences were tested to select one that depleted RhoE while not affecting Mad2. Specific sequences can be found in Table S2.

### Quantitative RT-PCR

RNA was prepared using the Qiagen RNAeasy kit according to the manufacturer’s instructions. 200 ng RNA was used to produce cDNA using the High-Capacity RNA-to-cDNA kit (Thermo Fisher Scientific) according to the manufacturer’s instructions. For quantitative RT-PCR, we used Power SYBR Green followed by analysis with a 7500 Real Time PCR system (Applied Biosystems). The primers used were DDR1: forward, 5′-CTGGTTAGTCTTGATTTCCC-3′; reverse, 5′-GGAAATCATTCCTGGCATTC-3′; GAPDH: forward, 5′-TTAAAAGCAGCCCTGGTGAC-3′; reverse, 5′-CTCTGCTCCTCCTGTTCGAC-3′.

### CDH1 CRISPR knockout

Two guide RNAs (gRNAs) targeting exon 1 within gene CDH1 were individually cloned into the LentiCRISPRv2 vector (Addgene) according to the manufacturer’s instructions and transduced together into cells, which were then selected using puromycin at 1–5 µg/ml followed by clonal selection for gene knockout. gRNAs used were 1, 5′-GCCGAGAGGCTGCGGCTCCA-3′, and 2, 5′-GCAGCAGCAGCAGCGCCGAG-3′.

### AFM

Indentations of cells by AFM were performed using a JPK NanoWizard-1 AFM (JPK) mounted on an inverted microscope (IX-81; Olympus). For our measurements, we used soft cantilevers with V-shaped tips (BioLever OBL-10 before experiments, nominal spring constant of 0.006 N/m; Bruker). The actual spring constant of the cantilever was calibrated using the thermal noise method implemented in the AFM software (JPK SPM). Before each experiment, the sensitivity of the cantilever was measured from the slope of force–distance curves that were acquired on glass. The day before experimentation, cells were plated onto 35-mm glass-bottom Petri dishes. On the day of the experiment, cells were incubated in MG132 (10 µM; Sigma-Aldrich) for 2 h before measurement to arrest cells in metaphase. Experiments were performed at room temperature, and cells were maintained in Leibovitz L15 medium (Life Technologies) supplemented with 10% FBS (Sigma-Aldrich) and 10 µM MG132. For each measurement, the cantilever was first aligned above a metaphase cell using the optical microscope. Then, force–distance curves were acquired over the center of the cell at the four vertices of a square with a 2-µm side. At each of these four positions, up to 10 curves were acquired with an approach speed of 2.5 µm/s and a target force of 2.5 nN. Experimental force–distance curves were postprocessed to compute an apparent elastic modulus. First, we determined the contact point between the cantilever tip and the cell using the method outlined by Crick and Yin (2007) implemented in Matlab (MathWorks). The indentation depth was then calculated by subtracting the cantilever deflection *d* from the piezo displacement beyond the contact point *z* (δ = *z* − *d*). The resultant force-indentation curves were then averaged over each position and fitted with the Sneddon model to calculate the apparent elasticity of each location probed in the cell (Sneddon, 1965). Curve fitting was restricted to indentation depths shallower than 800 nm to maximize contributions of the cortex to restoring force and minimize contributions from the cytoplasm.

### Centrosome tracking

Images were read into Matlab (R2017a) using the loci-tools java library (The Open Microscopy Environment). Centrosomes, represented by centrin1-GFP spots, were manually detected every *n* = 5–8 time points using a purpose-built Matlab graphical user interface. A *z*-projected image of the cell is shown at a given time point, and the user clicks the center of each centrin1-GFP signal. *z*-coordinates attributed to each selected spot are selected as the *z*-slice with the maximum mean intensity of the 500 × 500-nm *xy*-region surrounding the selected pixel. Spots at all other time points were localized using the location of the spots from the adjacent time point between itself and its nearest manual-detection time point. For example, if spots were manually detected at time points *t*_1_ and *t*_6_, spots at time point *t*_2_ were searched within a spherical mask, radius *r*, centered at each manually detected spot at time point *t*_1_. In a frame-wise manner, spots at time point *t*_3_ were then searched within spheres centered at the spots detected at time point *t*_2_. Time point *t*_4_, however, is closer to the manual-detection time point *t*_6_, and therefore spots at this time were searched within a sphere centered at the spots detected at time point *t*_5_ (rather than *t*_3_), which are themselves first searched within a sphere centered at the spots manually detected at time point *t*_6_. Here the mask radius *r* = (2 × *dt* × *v_avg_*) = (2 × 40 × 0.02) = 1.6 µm, where *dt* = 40 s is the time lapse, and *v_avg_* = 0.02 µm/s is the mean absolute speed of centrosome movement. 3D Gaussians were fitted to the detected spots to find subpixel spot center coordinates, and spots were tracked using KiT spot tracking software (Armond et al., 2016). The manual spot detection module is incorporated into KiT v.1.6.0 (available at https://github.com/cmcb-warwick).

### Statistical analysis

Appropriate statistical tests were applied using GraphPad Prism 5.0 or SPSS. In brief, Student’s *t* tests were used for comparisons between two groups. One-way ANOVA with Tukey post hoc test was used for comparison of three or more groups with one independent variable. Two-way ANOVA with Šidák post hoc test was used to evaluate the effects of two independent variables. Cell viability data were analyzed using two-way ANOVA with Šidák post hoc test by area under the curve. Rosette plots were created in R 3.3.1 using packages ggplot2 2.1, dplyr 0.5.0, and gridExtra 2.2.1.

### Online supplemental material

Fig. S1 describes the different methods used to induce centrosome amplification, MG132 treatment in RPE-1 and NIH-3T3 cells, and the localization of PCM components and HSET during mitosis. Fig. S2 provides information regarding the characterization of the different clones of CRIPR-Cas9 *CDH1^–^*^/–^ cells. It also describes the effects of PLK4 and TetR expression on centrosome clustering. Fig. S3 describes the effects of E-cadherin depletion of DDR1 protein and mRNA levels. Fig. S4 provides evidence that loss of E-cadherin in cancer epithelial cells has the same effect as shown in nontransformed cells. Fig. S5 shows that loss of RhoA GAPs does not impact clustering and that cortical contractility and astral MTs contribute for centrosome proximity. Fig. S6 shows that HSET levels at the spindle do not change with E-cadherin loss, and centrosome tracking reveals that loss of E-cadherin constricts centrosome movement in early stages of centrosome clustering but does not impact velocity of centrosome movement at later stages. Video 1 shows time-lapse imaging of binucleated MCF10A cells expressing centrin1-GFP and H2B-RFP undergoing bipolar division. Video 2 shows time-lapse imaging of binucleated MCF10A cells expressing centrin1-GFP and H2B-RFP undergoing multipolar division. Video 3 shows time-lapse imaging of individual centrosomes in binucleated MCF10A cells expressing centrin1-GFP. Video 4 shows time-lapse imaging of individual centrosomes in binucleated MCF10A CDH1^–/–^ cells expressing centrin1-GFP. Video 5 shows time-lapse imaging of individual centrosomes in binucleated MCF10A cells expressing centrin1-GFP after HSET depletion. Video 6 shows time-lapse imaging of individual centrosomes in binucleated MCF10A *CDH1^–^*^/–^ cells expressing centrin1-GFP after HSET depletion. Table S1 provides information regarding the cell lines, and Table S2 provides the siRNA sequences used in this study.

## Supplementary Material

Supplemental Materials (PDF)

Video 1

Video 2

Video 3

Video 4

Video 5

Video 6

Tables S1 and S2 (Excel)
