# Supplementary material for: Loss of E-cadherin provides tolerance to centrosome amplification in epithelial cancer cells
Source: J Cell Biol. 2018 Jan 2;217(1):195–209. doi: 10.1083/jcb.201704102 (PMC5748979; doi:10.1083/jcb.201704102)
Supplement: Supplemental Materials (PDF) [file JCB_201704102_sm.pdf]

Rhys et al., https://doi.org/10.1083/jcb.201704102

THE JOURNAL OF CELL BIOLOGY

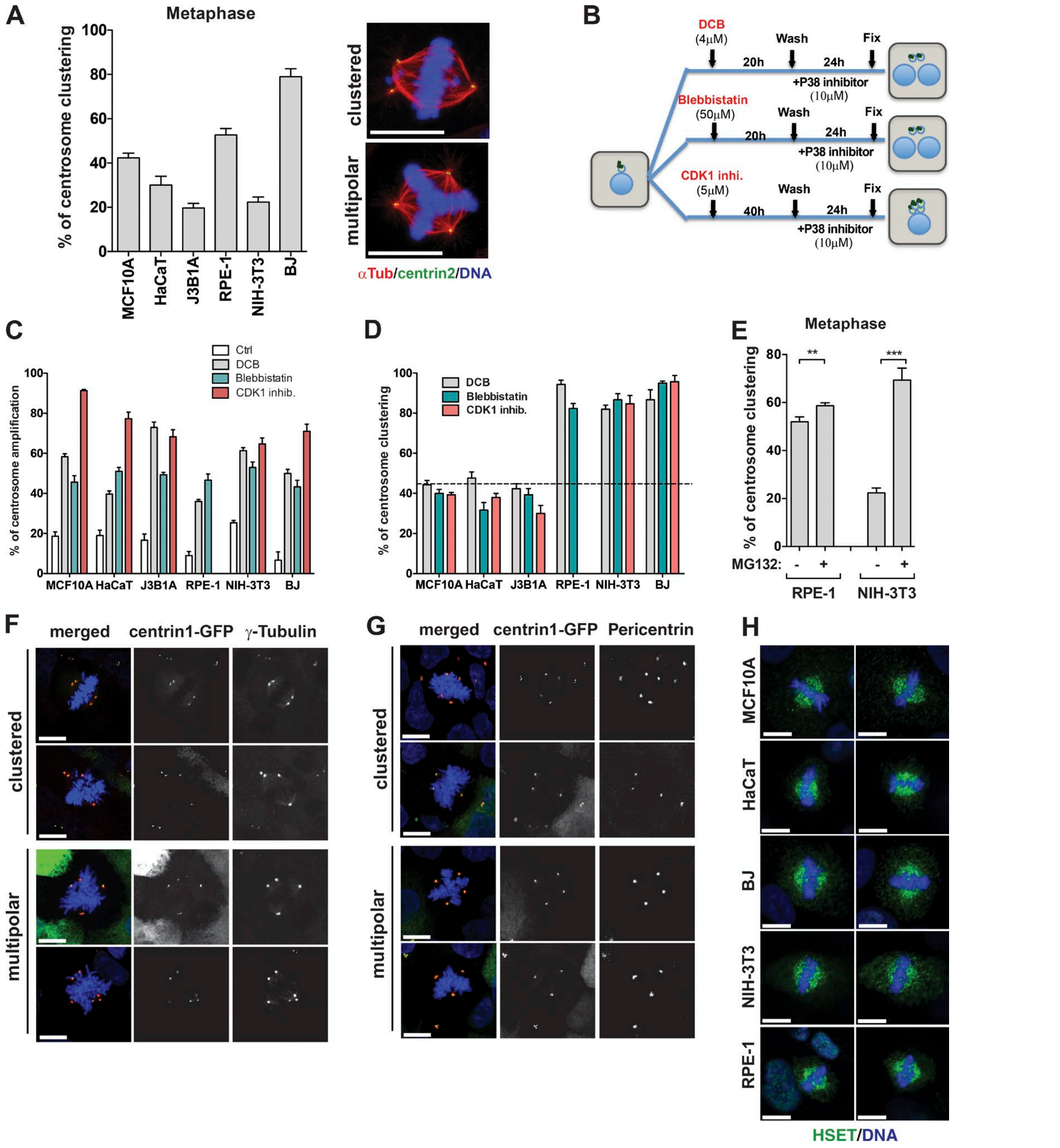

Figure S1. **Methods used to generate centrosome amplification in cell lines.** (A, left) Quantification of centrosome clustering in tetraploid cells at metaphase (n = 300). (Right) Images depicting examples of metaphase cells with extra centrosomes: bipolar clustered and multipolar. Cells were stained for microtubules (α-Tub, red), centrioles (centrin2, green), and DNA (blue). (B) Schematic representation of the different treatments to induce centrosome amplification. When required, p38 inhibitor was added to release cells from cell cycle arrest (10 µM, 24 h). (C) Quantification of centrosome amplification using different drug treatments. (D) Quantification of centrosome clustering in cytokinesis. *, RPE-1 cells that were treated with CDK1 inhibitor for 40 h arrested in interphase with fragmented nuclei and could not be analyzed. (E) Quantification of centrosome clustering in cells treated with 10 µM MG132 (4 h) at metaphase (n = 300). (F) MCF10A cells were stained for γ-Tubulin, (red), centrioles (centrin1-GFP, green), and DNA (blue). (G) Cells were stained for pericentrin (red), centrioles (centrin1-GFP, green), and DNA (blue). (H) Cells were stained for HSET (green) and DNA (blue). For all graphics, error bars represent mean ± SD from three independent experiments. **, $P < 0.01$; ***, $P < 0.001$. Bars, 10 µM.

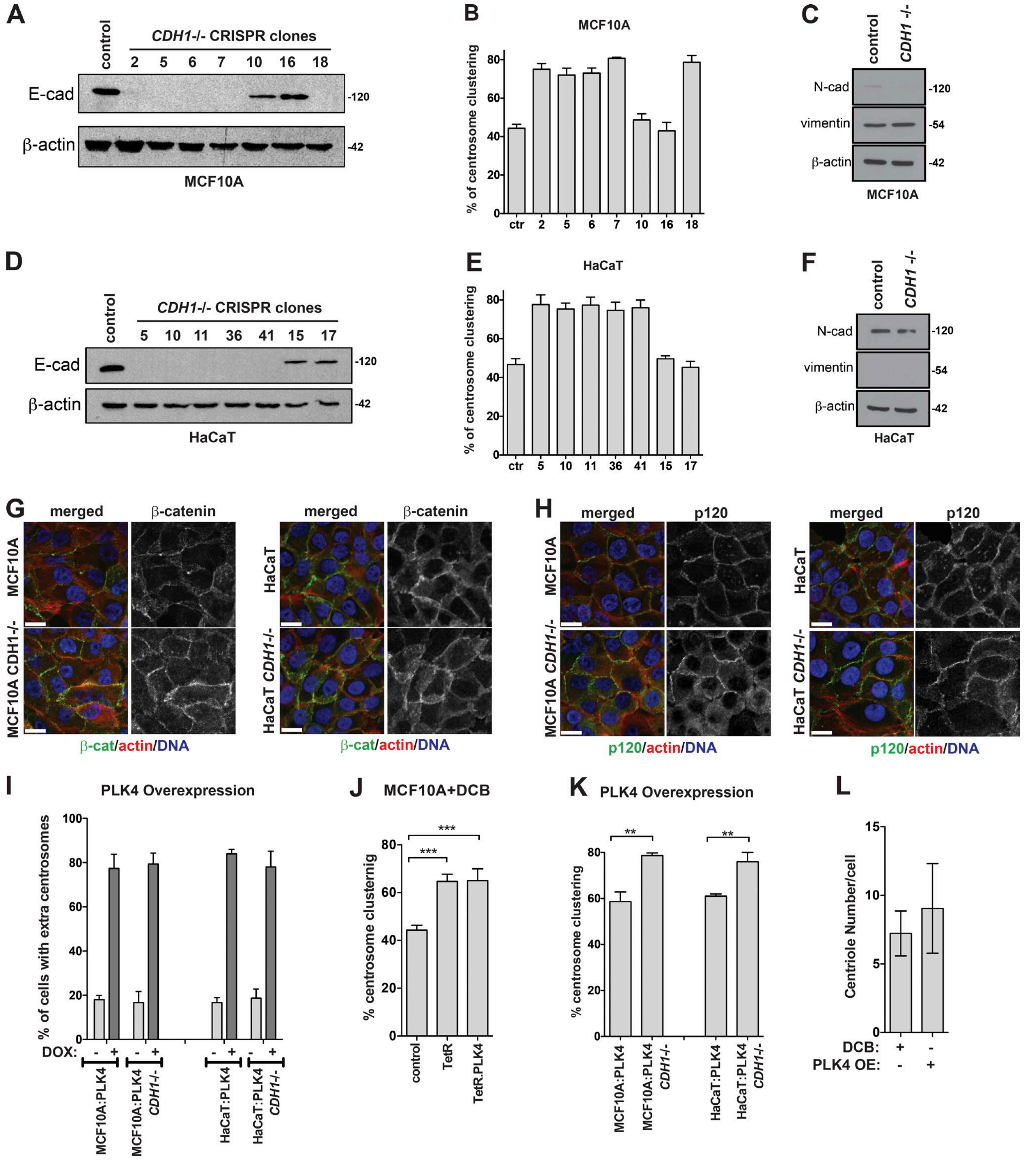

Figure S2. **Characterization of *CDH1*$^{-/-}$ CRISPR-Cas9 clones.** (A) Western blot analysis of the E-cadherin levels in MCF10A control and CRISPR-Cas9 *CDH1*$^{-/-}$ clones. (B) Quantification of centrosome clustering in cytokinesis in the CRISPR-Cas9 *CDH1*$^{-/-}$ clones after DCB treatment. (C) Western blot analysis showing that the levels of N-cadherin and vimentin do not change upon depletion of E-cadherin (no EMT was observed). (D) Western blot analysis of E-cadherin levels in HaCaT control and CRISPR-Cas9 clones. (E) Quantification of centrosome clustering in cytokinesis in the CRISPR-Cas9 *CDH1*$^{-/-}$ clones after DCB treatment. (F) Western blot analysis showing that the levels of N-cadherin and vimentin do not change upon depletion of E-cadherin (no EMT was observed). (G) Immunofluorescence of β-catenin in control and DDR1-depleted MCF10A and HaCaT cells. Cells were stained for β-catenin (green), F-actin (red), and DNA (blue). (H) Immunofluorescence of p120 catenin in control and DDR1-depleted MCF10A and HaCaT cells. Cells were stained for p120 (green), F-actin (red), and DNA (blue). (I) Quantification of centrosome amplification induced by transient PLK4 overexpression upon doxycycline (Dox) treatment (48 h). (J) Quantification of centrosome clustering upon DCB treatment in MCF10A cells expressing TetR alone or in combination with PLK4 (without Dox treatment). (K) Quantification of centrosome clustering in cytokinesis after PLK4 overexpression in control and *CDH1*$^{-/-}$ cells. (L) Number of centrioles per mitotic cell in cells treated with DCB or overexpressing PLK4. For all graphics, error bars represent mean ± SD from three independent experiments. **, $P < 0.01$; ***, $P < 0.001$. Bars, 20 µM.

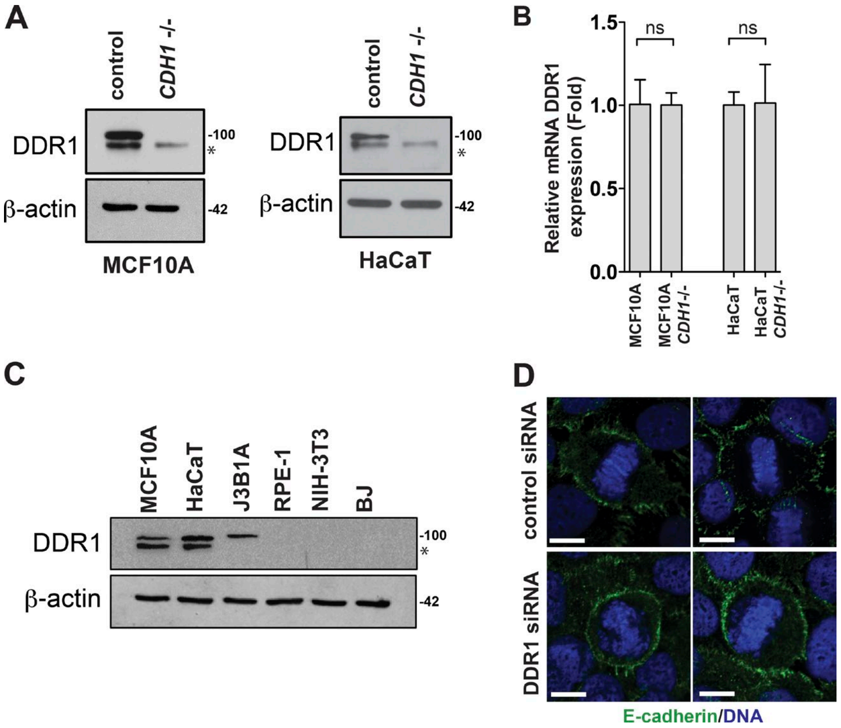

Figure S3. **DDR1 protein levels depend on E-cadherin expression.** (A) Western blot analysis of DDR1 levels in control and *CDH1*$^{-/-}$ MCF10A and HaCaT cells. *, a high-mannose immature form of DDR1. (B) Analyses of mRNA expression by qRT-PCR in in control and *CDH1*$^{-/-}$ MCF10A and HaCaT cells. (C) Western blot analysis of DDR1 levels in a panel of nontransformed cell lines. (D) Immunofluorescence of E-cadherin in control and DDR1-depleted HaCaT cells. Cells were stained for E-cadherin (green) and DNA (blue). Graphic error bars represent mean ± SD from three independent experiments. ns, not significant. Bars, 10 μM.

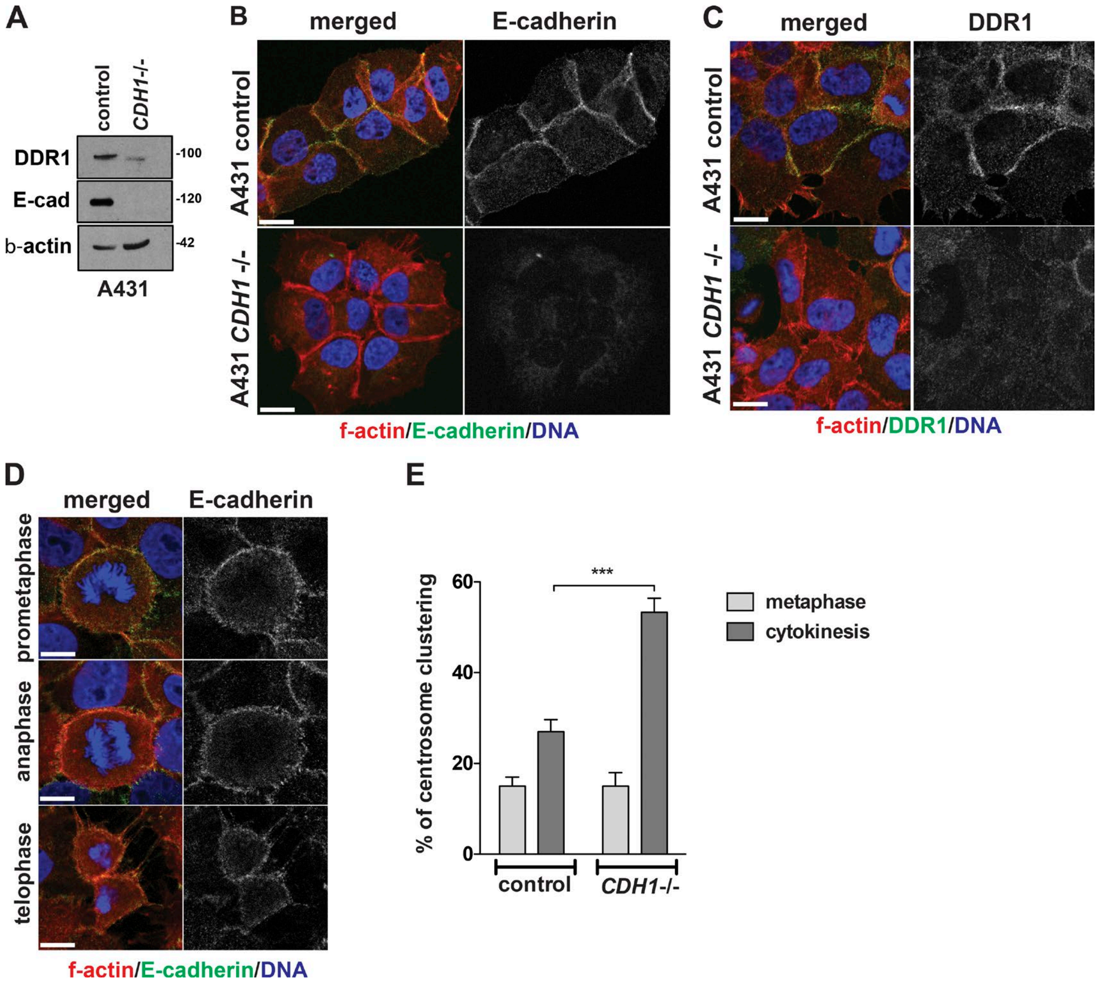

Figure S4. **Loss of E-cadherin using CRISPR-Cas9 knockdown in A431 squamous cancer cells leads to loss of DDR1 and defects in centrosome clustering.** (A) Western blot analysis of E-cadherin and DDR1 levels in control and *CDH1*$^{-/-}$ A431 cells. (B) Immunofluorescence of E-cadherin in control and *CDH1*$^{-/-}$ A431 cells. Cells were stained for F-actin (phalloidin, red), E-cadherin (green), and DNA (blue). Bars, 20 µM. (C) Immunofluorescence of DDR1 in control and *CDH1*$^{-/-}$ A431 cells. Cells were stained for F-actin (phalloidin, red), DDR1 (green), and DNA (blue). Bars, 20 µM. (D) Immunofluorescence of E-cadherin during mitosis. Cells were stained for F-actin (phalloidin, red), E-cadherin (green), and DNA (blue). Bars, 10 µM. (E) Quantification of centrosome clustering in metaphase and cytokinesis in control and *CDH1*$^{-/-}$ A431 cells. Graphic error bars represent mean ± SD from three independent experiments. ***, $P < 0.001$.

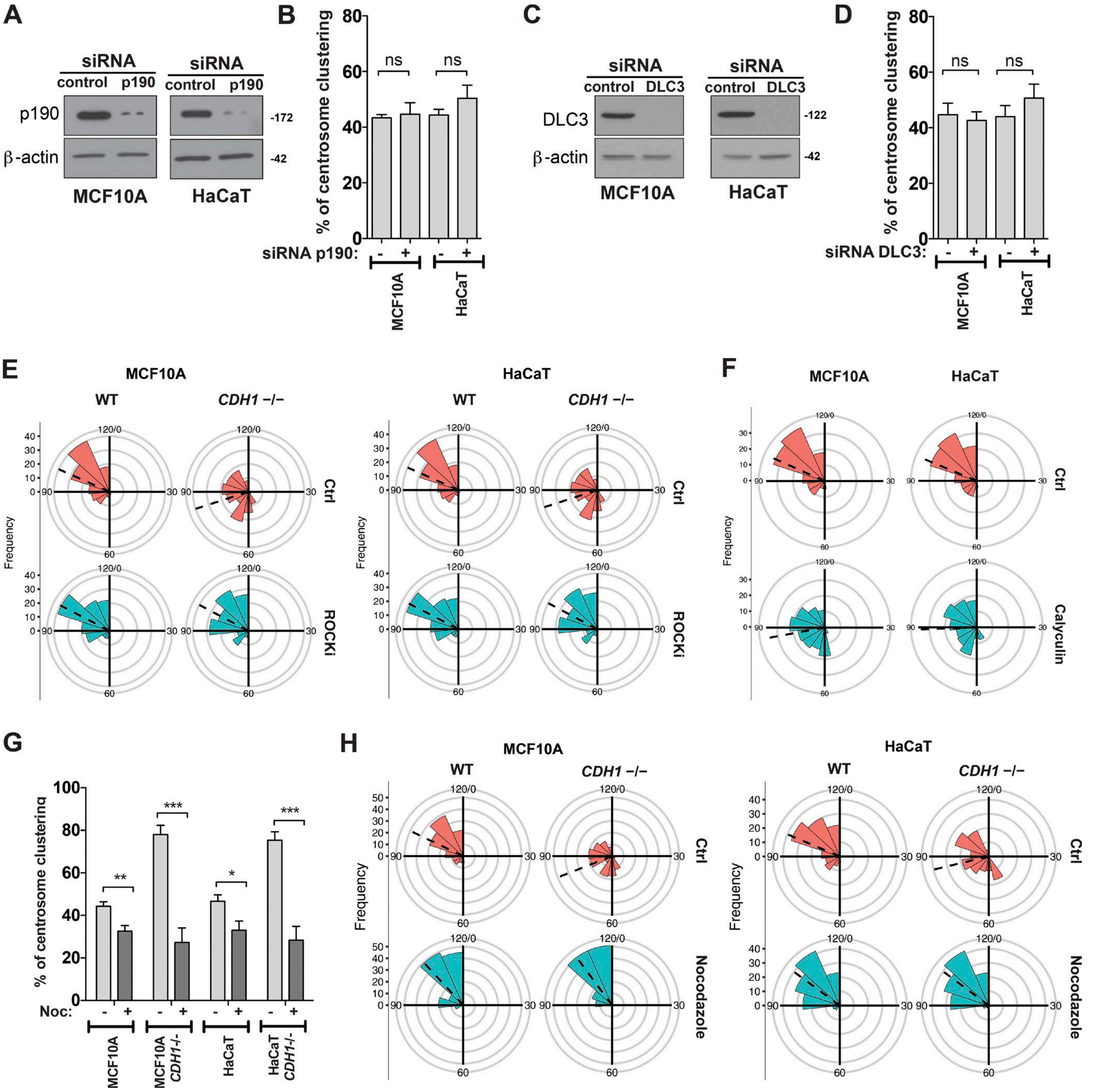

Figure S5. **Actomyosin contractility regulates centrosome movement and clustering.** (A) Western blot analysis of p190RhoGAP levels in MCF10A and HaCaT cells after siRNA depletion of p190RhoGAP. (B) Quantification of centrosome clustering in cytokinesis upon p190RhoGAP depletion (*n* = 150). (C) Western blot analysis of DLC3 levels in MCF10A and HaCaT cells after siRNA depletion of DLC3. (D) Quantification of centrosome clustering in cytokinesis upon DLC3 depletion (*n* = 150). (E) Rose plot showing the frequency of the angles measured in MCF10A and HaCaT cells (control and *CDH1*$^{-/-}$) upon treatment with the ROCK inhibitor Y-27632 (10 µM, 4 h). Dashed line represents the mean angle distribution. (F) Rose plot showing the frequency of the angles measured in MCF10A and HaCaT cells (control and *CDH1*$^{-/-}$) upon Calyculin A treatment (1 µM, 2 h). Dashed line represents the mean angle distribution. (G) Quantification of centrosome clustering in cytokinesis upon depletion of astral microtubules with low doses of nocodazole (5 nM, 3 h). (H) Rose plot showing the frequency of the angles measured in MCF10A and HaCaT cells (control and *CDH1*$^{-/-}$) upon treatment with low doses of nocodazole. Dashed line represents the mean angle distribution. Error bars represent mean ± SD from three independent experiments. *, P < 0.05; **, P < 0.01; ***, P < 0.001; ns, not significant.

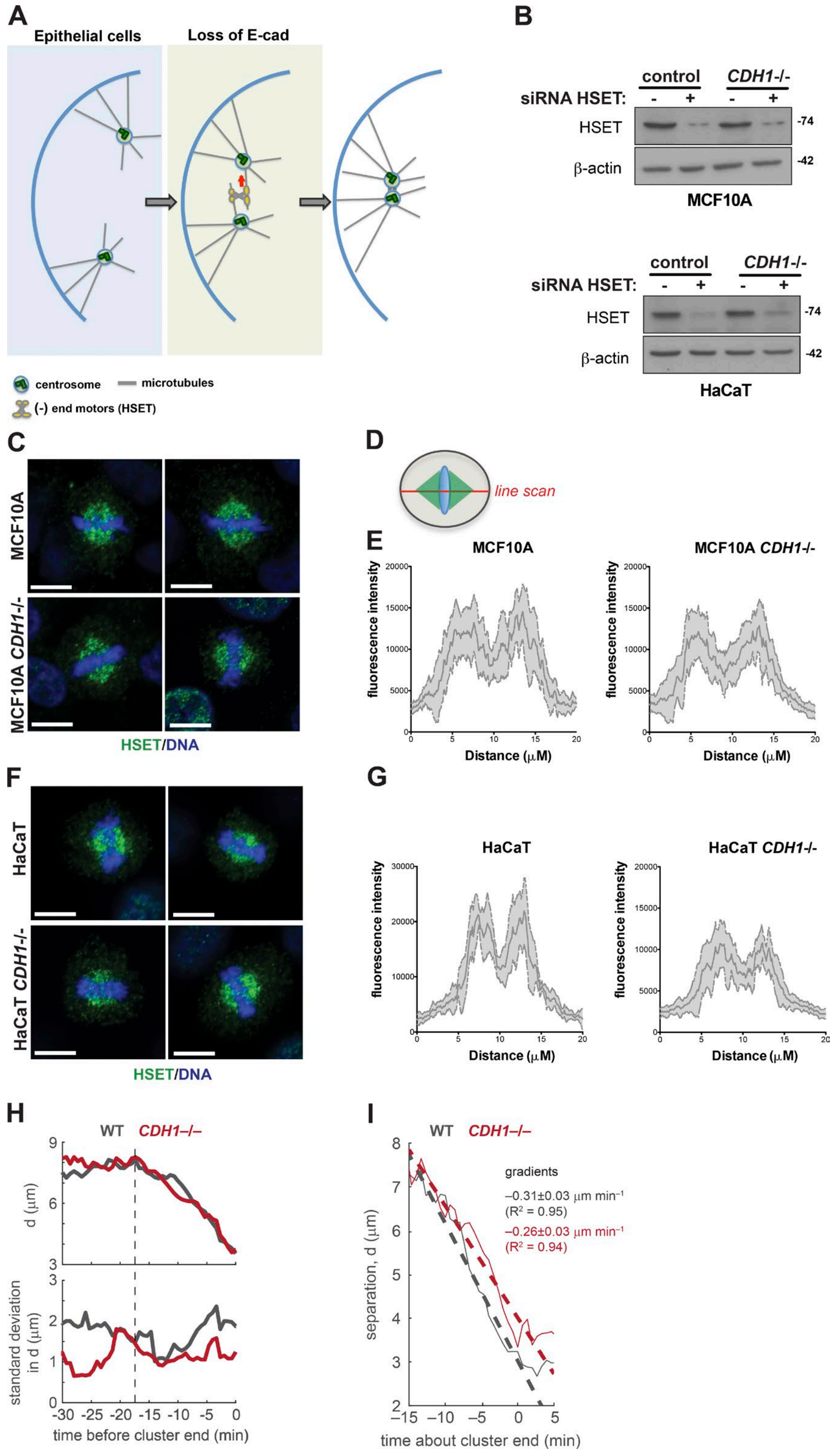

Figure S6. **HSET levels and mitotic spindle localization do not depend on E-cadherin expression.** (A) Schematic representation of our proposed clustering model. We propose that centrosome clustering by HSET requires centrosomes to be at close proximity. (B) Western blot analysis of HSET levels after siRNA depletion in HaCaT and MCF10A cells. (C) HSET localization in MCF10A cells (control and *CDH1*$^{-/-}$). Cells were stained for HSET (green) and DNA (blue). (D) Schematic representation of the line scan used to quantify HSET fluorescence intensity. (E) Graphics represent fluorescent intensity (arbitrary units, AU) over the length of the spindle. Dashed lines represent SD (WT, n = 12; CDH1$^{-/-}$, n = 14). (F) HSET localization in HaCaT cells (control and *CDH1*$^{-/-}$). Cells were stained for HSET (green) and DNA (blue). (G) Graphics represent fluorescent intensity (AU) over the length of the spindle. Dashed lines represent SD (WT, n = 7; CDH1$^{-/-}$, n = 12). (H) The population mean of all trajectories depicting centrosome distance, *d*, over time showing the biphasic nature of centrosome clustering. The vertical dashed line at –17.5 min separates the two phases: search and capture followed by the motorized clustering phase. Cluster completion was defined as the time point at which separation stabilized. (I) Best-fit (dashed) lines through the median centrosome separation during motorized clustering in MCF10A WT (gray; R^2 = 0.95) and MCF10A *CDH1*$^{-/-}$ (red; R^2 = 0.94) cells. Lines, representing clustering speed, have gradients –0.31 ± 0.03 and –0.26 ± 0.03 µm/min in WT and *CDH1*$^{-/-}$ cells, respectively. Lines were fitted using least absolute residuals. Bars, 10 µM.

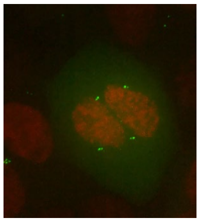

Video 1. **Time-lapse imaging of binucleated MCF10A cells with extra centrosomes expressing centrin1-GFP and H2B-RFP dividing in a bipolar fashion (centrosome clustering).** Cells were imaged every 40 s for 54 min.

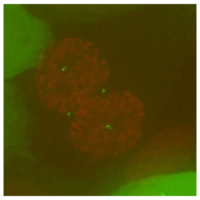

Video 2. **Time-lapse imaging of binucleated MCF10A cells with extra centrosomes expressing centrin1-GFP and H2B-RFP dividing in a multipolar fashion.** Cells were imaged every 40 s for 56 min.

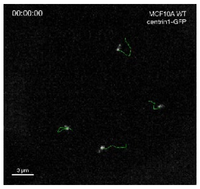

Video 3. **Time-lapse imaging of binucleated MCF10A cells with extra centrosomes expressing centrin1-GFP.** Green and red lines represent centrosome trajectories during the eight time points (every 40 s) before and after each time frame, respectively. Cells were imaged every 40 s for 1 h and 8 min.

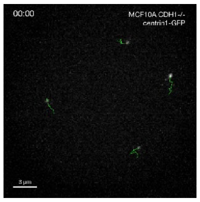

Video 4. **Time-lapse imaging of binucleated MCF10A** *CDH1*$^{-/-}$ **cells with extra centrosomes expressing centrin1-GFP.** Green and red lines represent centrosome trajectories during the eight time points (every 40 s) before and after each time frame, respectively. Cells were imaged every 40 s for 28 min.

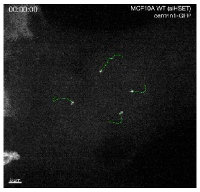

Video 5. **Time-lapse imaging of binucleated MCF10A cells with extra centrosomes expressing centrin1-GFP after HSET depletion by siRNA (48 h).** Green and red lines represent centrosome trajectories during the eight time points (every 40 s) before and after each time frame, respectively Cells were imaged every 40 s for 1 h and 2 min.

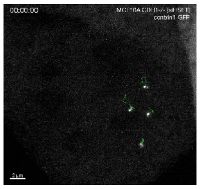

Video 6. **Time-lapse imaging of binucleated MCF10A** *CDH1*$^{-/-}$ **cells with extra centrosomes expressing centrin1-GFP after HSET depletion by siRNA (48 h).** Green and red lines represent centrosome trajectories during the eight time points (every 40 s) before and after each time frame, respectively. Cells were imaged every 40 s for 1 h and 6 min.

Provided online are two tables in Excel. Table S1 provides information regarding the cell lines and Table S2 provides the siRNA sequences used in this study.
